# The human respiratory tract microbial community structures in healthy and cystic fibrosis infants

**DOI:** 10.1038/s41522-020-00171-7

**Published:** 2020-12-15

**Authors:** Marie-Madlen Pust, Lutz Wiehlmann, Colin Davenport, Isa Rudolf, Anna-Maria Dittrich, Burkhard Tümmler

**Affiliations:** 1 Clinic for Paediatric Pneumology, Allergology, and Neonatology, Hannover Medical School, Hannover, Germany; 2 Biomedical Research in Endstage and Obstructive Lung Disease Hannover (BREATH), German Center for Lung Research, Hannover Medical School, Hannover, Germany; 3 Research Core Unit Genomics, Hannover Medical School, Hannover, Germany

**Keywords:** Metagenomics, Microbiome, Microbial ecology

## Abstract

The metagenome development of the human respiratory tract was investigated by shotgun metagenome metagenomic sequencing of cough swabs from healthy children and children with cystic fibrosis (CF) between 3 weeks and 6 years of age. A healthy microbial community signature was associated with increased absolute abundances in terms of bacterial–human cell ratios of core and rare species across all age groups, with a higher diversity of rare species and a tightly interconnected species co-occurrence network, in which individual members were found in close proximity to each other and negative correlations were absent. Even without typical CF pathogens, the CF infant co-occurrence network was found to be less stable and prone to fragmentation due to fewer connections between species, a higher number of bridging species and the presence of negative species correlations. Detection of low-abundant DNA of the CF hallmark pathogen *Pseudomonas aeruginosa* was neither disease- nor age-associated in our cohort. Healthy and CF children come into contact with *P. aeruginosa* on a regular basis and from early on.

## Introduction

Until recently the human lower airways have been considered to be sterile and consequently most studies investigated the lung microbiology in conditions of acute infections or chronic lung disease, such as cystic fibrosis (CF)^1–10^. Meanwhile, we know that the lower respiratory tract microbiome is shaped by transient microbial colonisation based on regular migration of microorganisms from the upper to the lower respiratory tract through microaspiration and inhalation with the subsequent clearance of invaders by host defence mechanisms^11–14^.

However, to our knowledge information is not available on the healthy lower respiratory tract metagenome, and its development in terms of microbial biodiversity and species co-occurrence networks in the early years of life.

Hence, we set up a microbial metagenome study based on shotgun metagenomic sequencing and collected cough swabs of healthy (*n* = 52) children with no history of pulmonary disease, and children with CF (*n* = 41) between 3 weeks and 6 years of age. CF is the most common severe autosomal recessive genetic disorder in Caucasians with chronic bacterial airway infections being the major life-limiting morbidity^15–18^. A personalised metagenome signature with many low-abundant and a few dominant pulmonary pathogens, such as *Staphylococcus aureus* and *Pseudomonas aeruginosa* has been described for adult CF patients and patients with end-stage lung disease^19–21^. It is currently unknown when this typical CF metagenome signature emerges. In addition, the literature suggests that the first time point of *P. aeruginosa* observation in culture corresponds to the first time point of *P. aeruginosa* airway colonisation^22,23^. We have observed in a previous metagenome study that *P. aeruginosa*-DNA was present in the respiratory secretions of all pancreatic insufficient (PI) CF patients aged 6 years or older in at least minute amounts, while *P. aeruginosa* was not detectable by culture-dependent diagnostics^19^. Here again, it remains unknown when the CF children come into contact with the CF hallmark pathogen for the first time.

So, on the one hand, we set out to investigate the early development of the healthy and CF respiratory tract metagenome in terms of microbial biodiversity and species co-occurrence patterns. On the other hand, we aimed to identify the first time point at which a typical CF signature becomes apparent and *P. aeruginosa*-DNA can be isolated from cough swabs of CF patients for the first time.

In order to undertake these investigations, we utilised a deep sequencing strategy with single-end and short reads (75 base pairs, bp), which were obtained from human and microbial DNA of patients’ cough swabs. The human DNA was exploited as natural spike-in control to normalise the bacterial reads to human reads, and obtain insights into the absolute abundance patterns of bacterial airway inhabitants^19^. The absolute abundance estimations enabled us to approach a broad range of statistical tools for comparative microbial community analyses, including ordination, clustering and network analysis^24–30^. We probed the maximum number of bacterial genome positions by generating single-end instead of paired-end reads and consequently, were able to cover the rare species of the airway habitat in our metagenome investigation.

We found that the early healthy and CF airway metagenomes were not distinct in alpha and beta diversity of core and rare species in the first 3 years of life. Supervised and unsupervised clustering algorithms failed to identify a CF-specific microbial community profile in newborns and preschool children. However, the early healthy and CF airway metagenomes were distinct in the absolute abundance of core and rare species, and the evolution of their species co-occurrence networks. Surprisingly, trace amounts of *P. aeruginosa*-DNA were stochastically detected with equal shares in both cohorts, healthy and CF.

## Results

### Study participants

CF patients were sampled exclusively at our outpatient clinic, while healthy controls were sampled at various locations in Hannover, Germany. We collected cough swabs from 41 patients with CF and 52 healthy controls between 0 and 6 years of age (Table 1). At sampling, children had a median age of 26 months (CF) vs. 11 months (healthy controls). Our study was conducted 3 years after the introduction of the CF newborn screening in Germany (2016). Thus, we included a longitudinal cohort of 11 CF patients identified by newborn screening from whom we collected 36 consecutive samples with a mean number of 3.2 samples per patient over a period of 13 months (Supplementary Table 1).

**Table 1:** Metadata of healthy and CF-diagnosed participants.

Variable of interest	Healthy cohort	Patient cohort
Number of subjects (*n*)	52	41
Number of subjects in longitudinal cohort	0	11
Age^a^		
Median age at sample collection in months (age range)	11 (1–75)	26 (0–82)
Median age at diagnosis in months (age range)	Not applicable	8 (0–39)
Number of subjects in age groups: 0, 1–3, 4–6 years	28, 9, 15	5, 20, 16
Gender^b^		
Number of female subjects (in %)	21 (40%)	14 (34%)
Number of male subjects (in %)	31 (60%)	27 (66%)
Number of samples collected at different locations		
Kindergarten	17 (33%)	0
Local paediatrician (preventive medical check-up)	16 (31%)	0
Parent–child groups	19 (36%)	0
CF outpatient clinic	0	41 (100%)
First clinical indication for CF		
CF newborn screening	Not applicable	15 (37%)^c^
Family history	Not applicable	4 (10%)
Meconium ileus	Not applicable	4 (10%)
Gastrointestinal and/or pulmonary symptoms	Not applicable	18 (43%)
Pancreatic state		
Pancreatic insufficient (PI)	0	33 (80%)
Pancreatic sufficient (PS)	52 (100%)	8 (20%)
Class of *CFTR* mutation		
II/II	Not applicable	20 (49%)
I/I	Not applicable	1 (2%)
I/II	Not applicable	13 (32%)
IV/other^d^ or V/other^d^	Not applicable	7 (17%)

^a^Age (in months) was different between CF and healthy (Wilcoxon *p* value = 0.003, *r* = 0.31, CI = 0.12–0.48).

^b^Gender distribution of healthy and CF was not different (Fisher’s exact test for count data, *p* value > 0.05).

^c^Eleven of the 15 newborns were recruited for the longitudinal study. Only one sample is currently available from the other four infants.

^d^Known class I, II or III PI mutation

### Quality control measures

DNA background contamination can profoundly affect metagenome analyses of low-biomass environments^31–35^, precautions in terms of appropriate negative controls must therefore be implemented. Contamination was continuously tracked by preparing, and sequencing blank cotton swabs and water controls in parallel with patient samples. Analyses of these controls revealed that on average 96% of DNA reads per sample either aligned to the human reference genome or were of low quality leading to exclusion (Supplementary Fig. 1). We observed a typical microbial pattern in these experimental controls consisting of *Cutibacterium acnes*, *Ralstonia pickettii* and *Achromobacter xylosoxidans*. We could ascribe the ‘contamination’ of *A. xylosoxidans* (accession number: CP006958.1, *Achromobacter xylosoxidans* NBRC) to erroneous inclusion of sequence adapters into the reference genome. Neither *P. aeruginosa* nor other typical inhabitants of the respiratory tract were detected.

### The early development of microbial biodiversity in the airway metagenome from birth to 6 years of age

#### Relative and absolute abundance of species

**Fig. 1:**
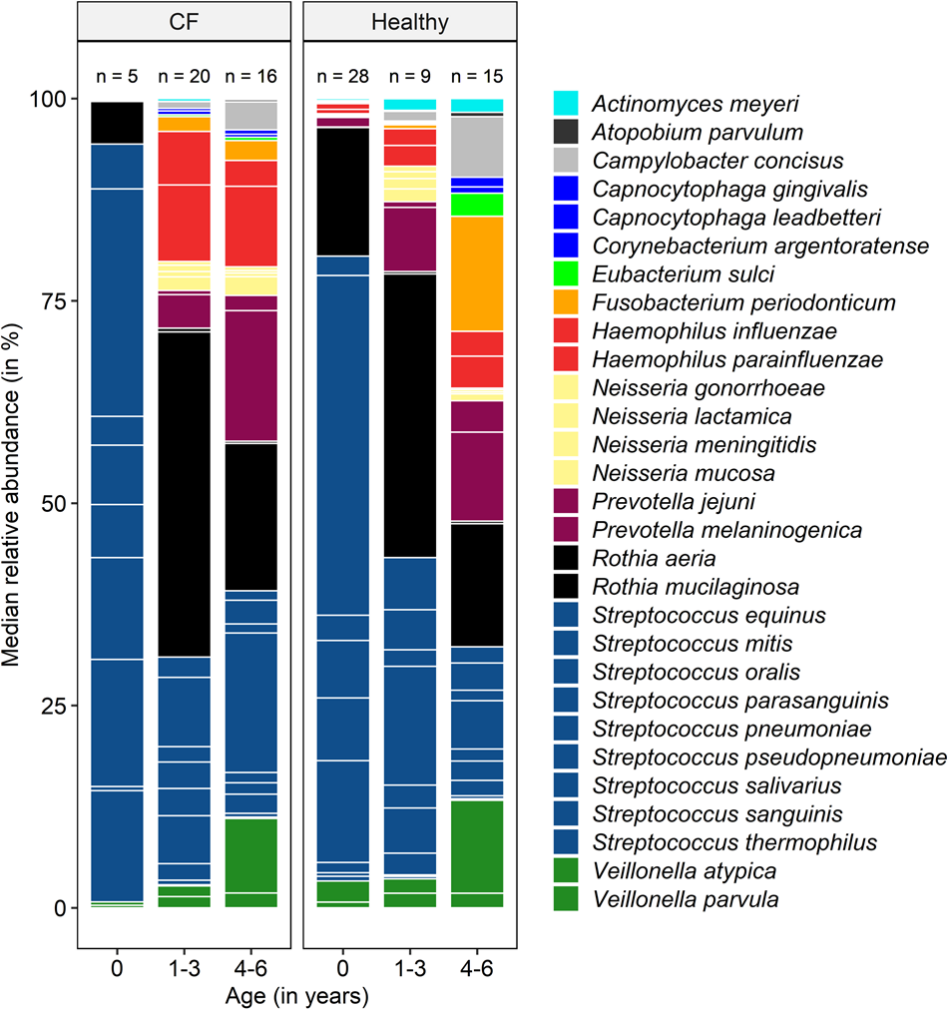
Stacked barplot of median relative abundance (in %) across age groups in healthy and CF children. Relative abundance was calculated from bacterial to human cell ratios. The bars and legend are sorted alphabetically. The colours represent taxonomic classification at genus level. The white lines, which are separating blocks of the same colour depict median relative abundances of species. The order of species per colour bar is alphabetic. The numerical data and statistically significant differences based on species levels are listed in Supplementary Table 2. The total number of children (*n*) in each group is stated on top of the bar.

In the first year of life, the healthy and CF respiratory tract was dominated by bacteria from the genus Streptococcus with relative abundances of 77% and 89%, respectively (Fig. 1 and Supplementary Table 2). In both groups, the most abundant non-*Streptococcus* species was *Rothia mucilaginosa* with relative abundances of 16% in healthy and 10% in CF infants (Fig. 1 and Supplementary Table 2). While *Prevotella melaninogenica* and *Prevotella jejuni* were absent in the first year of CF infants (0%, Supplementary Table 2), those species started to emerge already slightly in the healthy respiratory tract metagenome (1.3%, Supplementary Table 2), though the difference was not found to be statistically significant. Minor amounts of bacteria from the genus Veillonella were observed in both cohorts from the very beginning (Supplementary Table 2). In the first 4 years of life, the relative abundance patterns of core species were similar between CF and healthy children. Overall, relative abundances of *Streptococcus* spp. decreased with age and relative abundances of *P. melaninogenica*, *P. jejuni*, *Veillonella parvula*, *Veillonella atypica*, *Neisseria* spp. and *Haemophilus* spp. increased (Fig. 1 and Supplementary Table 2). After the age of 4 years, the number of species with significantly higher relative abundances in healthy children increased. No bacterial species were detected with higher relative abundances in CF compared to healthy infants.

**Fig. 2:**
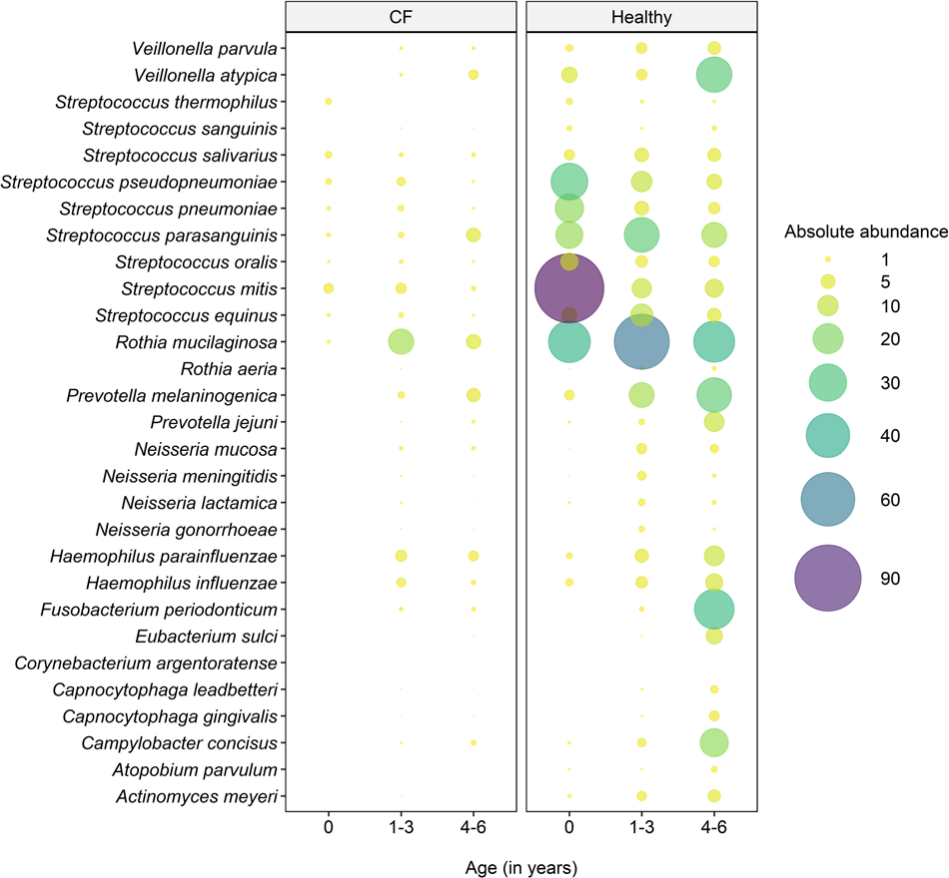
Bubble plot of the median absolute abundance of core species in the respiratory tract of CF children (left) and healthy children (right) across age groups. CF children display lower absolute abundances of all core species in the respiratory tract compared to healthy controls across all age groups. The absolute abundance was calculated as described by Losada et al.^19^, where the length of the diploid human genome is divided by a million to account for the bacterial count scale. The quotient is multiplied by the normalised bacterial read count (normalised to a million reference base pairs) and the final product is divided by the human read count. The statistically significant differences in absolute abundances are confirmed in Supplementary Fig. 2. In the CF cohort, there were 5 infants below the age of one, 20 between 1 and 3 years of age and 16 children between 4 and 6 years of age. In the healthy cohort, there were 28 infants below the age of one, 9 children between 1 and 3 years of age and 15 preschool children were 4 and 6 years of age.

When assessing absolute abundances^19^, we could demonstrate that healthy children consistently harboured more bacterial cells per human cell of all core species than CF children, regardless of age (Fig. 2 and Supplementary Fig. 2).

#### Alpha diversity

We analysed Shannon diversity indices (SDI) of the core (the 95% most abundant species) and rare (the 5% least abundant) species across different age groups of our healthy and CF probands. No significant difference in core species diversity was detected in the first 3 years of life (Supplementary Fig. 3, left). By the age of 4 years, a significant difference in core species diversity was observed. While the diversity of core species in healthy children constantly increased over time with a significant difference of diversity between healthy infants (first year of life) and healthy preschool children (fourth to sixth year of life), the diversity of core species in the CF metagenome remained almost unchanged (Supplementary Fig. 3, left). Investigations of the 5% least abundant species in the respiratory tract metagenome of healthy and CF infants revealed no significant differences between healthy and CF children with age. In both cohorts, however, the interquartile range was large, suggesting high age-independent variability of rare species diversity in health and CF (Supplementary Fig. 3, right).

#### Beta diversity

**Fig. 3:**
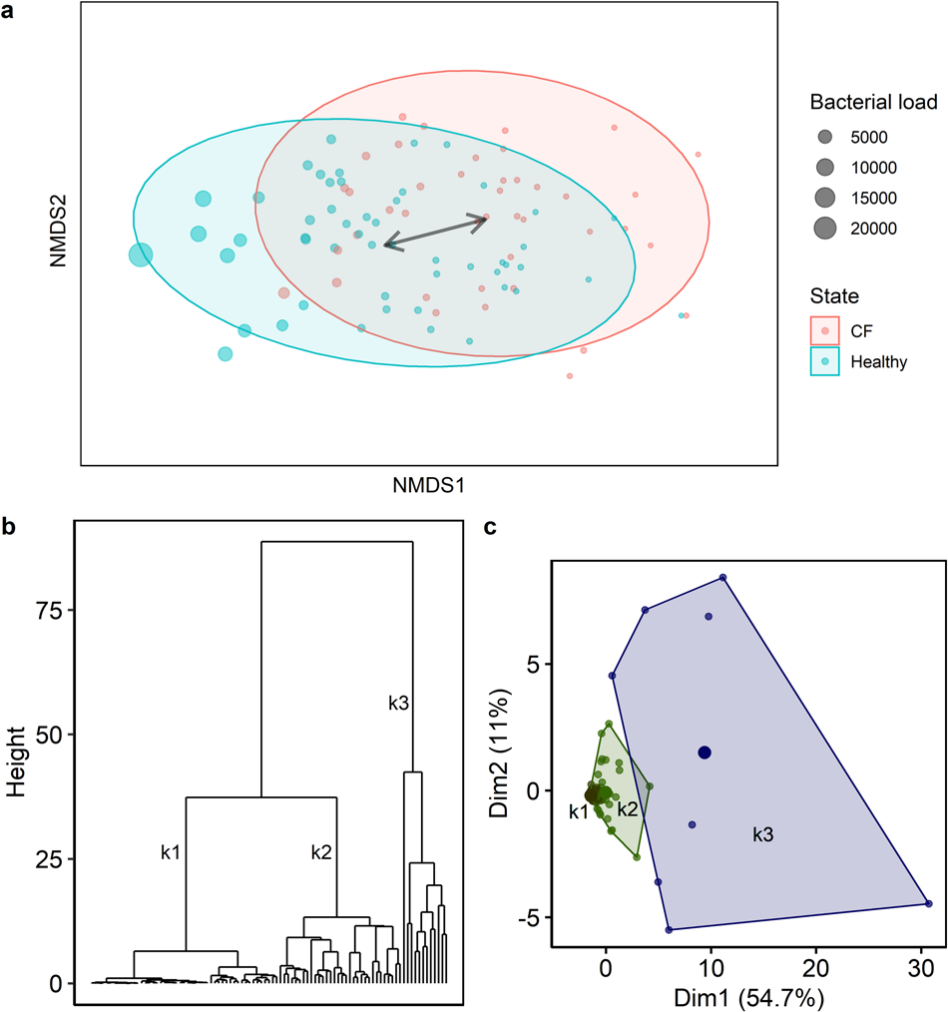
Clustering analyses to identify known and unknown patterns, which influence microbial community profiles in children. **a** Non-metric multidimensional scaling based on Bray–Curtis dissimilarity matrices identified massive overlap between healthy and CF microbial community profiles. The bacterial load (absolute abundance) indicates the number of bacterial cells per human cell. **b** Hierarchical clustering (Ward’s clustering algorithm) based on Euclidean distances revealed three main groups of microbial community profiles in children between 0 and 6 years of age. **c** The group pattern was confirmed by principal component analysis (PCA). The PCA plot contains the first and second principal components as *x*- and *y*-axis, respectively. All core species contribute equally to the variance observed in the PCA, for example *Streptococcus oralis* explains 4.8% of the variance, *R. mucilaginosa* 4.7%, *V. parvula* 4.7%, *Streptococcus parasanguinis* 4.7%, *Fusobacterium periodonticum* 4.6% and so on (Supplementary Table 3).

**Fig. 4:**
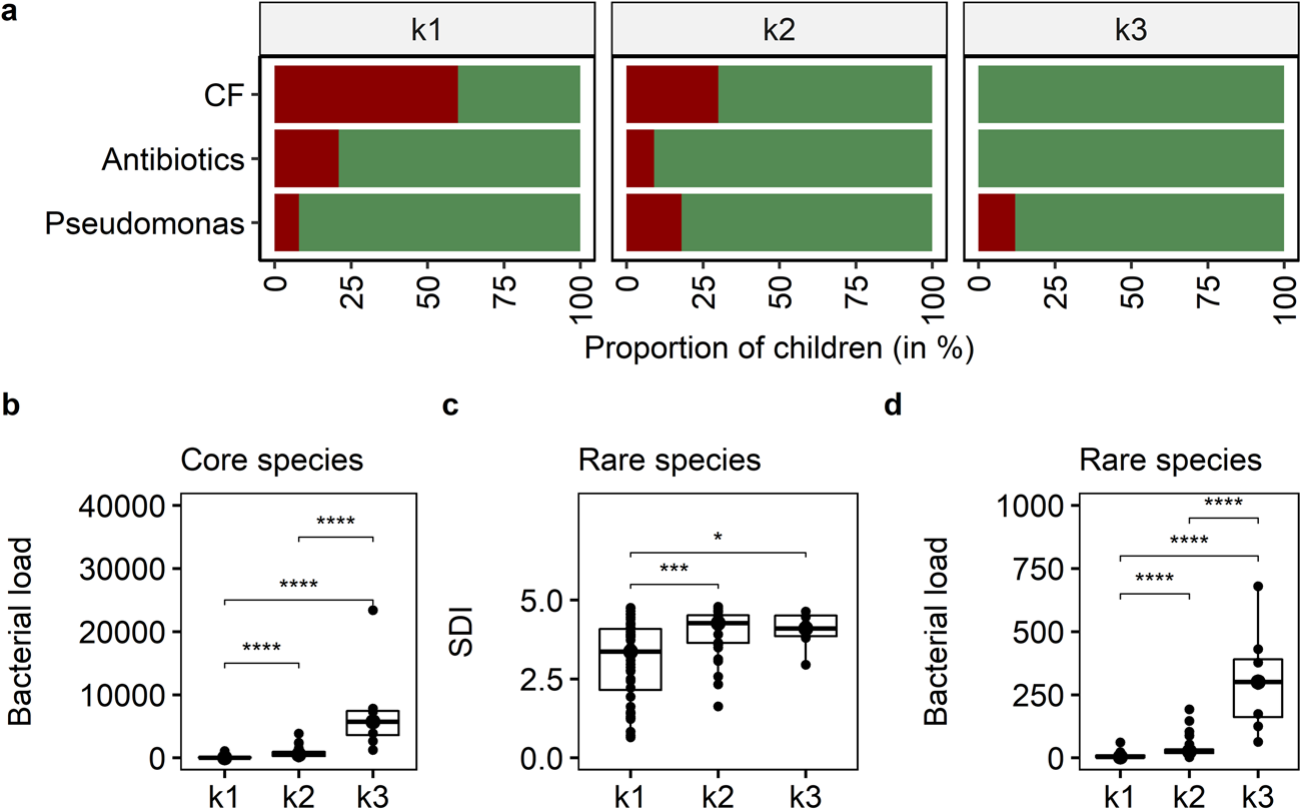
Cluster characteristics based on clinical and microbial community data. **a** Proportion of children in each cluster who were diagnosed with CF (red), considered as *P. aeruginosa*-DNA positive (red), received antibiotics in the month of sampling (red) and those who did not (green). **b** Bacterial load of core species in terms of bacterial cells per human cell (Kruskal–Wallis *p* value < 0.0001, e2 = 0.60, CI = 0.45–0.72). **c** Shannon diversity indices (SDI) of rare species (Kruskal–Wallis *p* value = 0.0005, e2 = 0.17, CI = 0.05–0.34) and **d** bacterial load of rare species across the three cluster groups (Kruskal–Wallis *p* value < 0.0001, e2 = 0.55, CI = 0.39–0.69). Pairwise comparison was done using the Conover–Iman test and Benjamini–Hochberg adjustment (pairwise *p* values are given in the diagram with *p < 0.05, **p < 0.01, ***p < 0.001, ****p < 0.0001). The centre line of the boxplot depicts the median (50th percentile). The lower and upper boundary of the box represent the first (25th percentile) and third (75th percentile) quartile, and hence define the interquartile range (IQR). Whiskers extend from the box to the largest/smallest non-outlier data point (1.5 × IQR).

Next, we performed non-metric multidimensional scaling (nmds) to assess the effect of clinical and environmental variables on the core respiratory tract community structure. Therefore, we fitted known parameters onto the ordination, including age group and disease state (CF vs. healthy), season of sampling, antimicrobial therapy in the month of sample collection, sampling of siblings and the presence of *P. aeruginosa*-DNA. Whereas the core community structure was slightly influenced by age group and disease state (Table 2), other aspects had no significant effect on the core community structures (Table 2). Instead, a massive overlap between microbial community profiles of healthy and CF children was visible (Fig. 3a). However, a spare sampling test (Hopkins statistic, *H*)^36^ was applied to measure the cluster tendency of the dataset, and a strong non-random cluster structure was observed (*H* = 0.85). Since no cluster behaviour was apparent by investigating the clinical or environmental variables known to us (Table 2), we approached unsupervised hierarchical clustering and principal component analysis (Supplementary Table 3) to identify the hidden clusters of microbial community profiles in all children regardless of disease state. Unsupervised clustering revealed three distinct groups (k1–k3) of microbial community profiles between 0 and 6 years of age (Fig. 3b, c). Between those three profiles, we compared *P. aeruginosa*-DNA detection, disease state and antibiotic therapy. Both k1 and k2 groups comprised healthy and CF children with and without *P. aeruginosa*-DNA detection, and children receiving antibiotics and antibiotic-free children (Fig. 4a). Group k1 and k2 overlapped, but k1 comprised less healthy children and more children receiving antibiotics than k2. In k3, all children were healthy and reported no antibiotic usage. SDI of the three groups were similar for the core metagenome. For the rare species, however, we found significantly lower diversity in group k1 relative to k2 and k3 (Fig. 4b). When considering bacterial abundance of the core and rare species, group k1 showed the lowest bacterial load, followed by groups k2 and k3 (Fig. 4c, d).

**Table 2:** Non-metric multidimensional scaling based on the Bray–Curtis dissimilarity matrices^a^.

Parameters	Goodness of fit, *r*^2^	Goodness of fit, *p*
Antimicrobial therapy	0.05	0.11
Siblings	0.02	0.99
Season of sampling	0.06	0.15
Disease state	0.16	0.001***
*P. aeruginosa*-DNA	0.002	0.78
Age group	0.08	0.01*
Shannon diversity (core species)	0.12	0.006**
Shannon diversity (rare species)	0.43	0.001***
Absolute abundance (core species)	0.32	0.001***
Absolute abundance (rare species)	0.39	0.001***

^a^A good representation in reduced dimensions was observed (stress = 0.07). The significance of known factors fitted to the ordination was assed using a permutation test (*n* = 999, R vegan package, envfit).

### Establishments of species co-occurrence networks in the first 3 years of life

We investigated species co-occurrence development of the 99% most abundant species in the first 3 years of life in health and CF by ecological network analysis^37^, with a continuous graph layout algorithm^38^. We focused on the following three parameters: degree centrality, closeness centrality and betweenness centrality. Degree centrality measures the numbers of connections of a node. Closeness centrality calculates the shortest distance of a node to all other nodes in the network^37^, where a high value refers to a more central node. Betweenness centrality measures how often a node is bridged by the shortest pathway of two other nodes^37^.

**Fig. 5:**
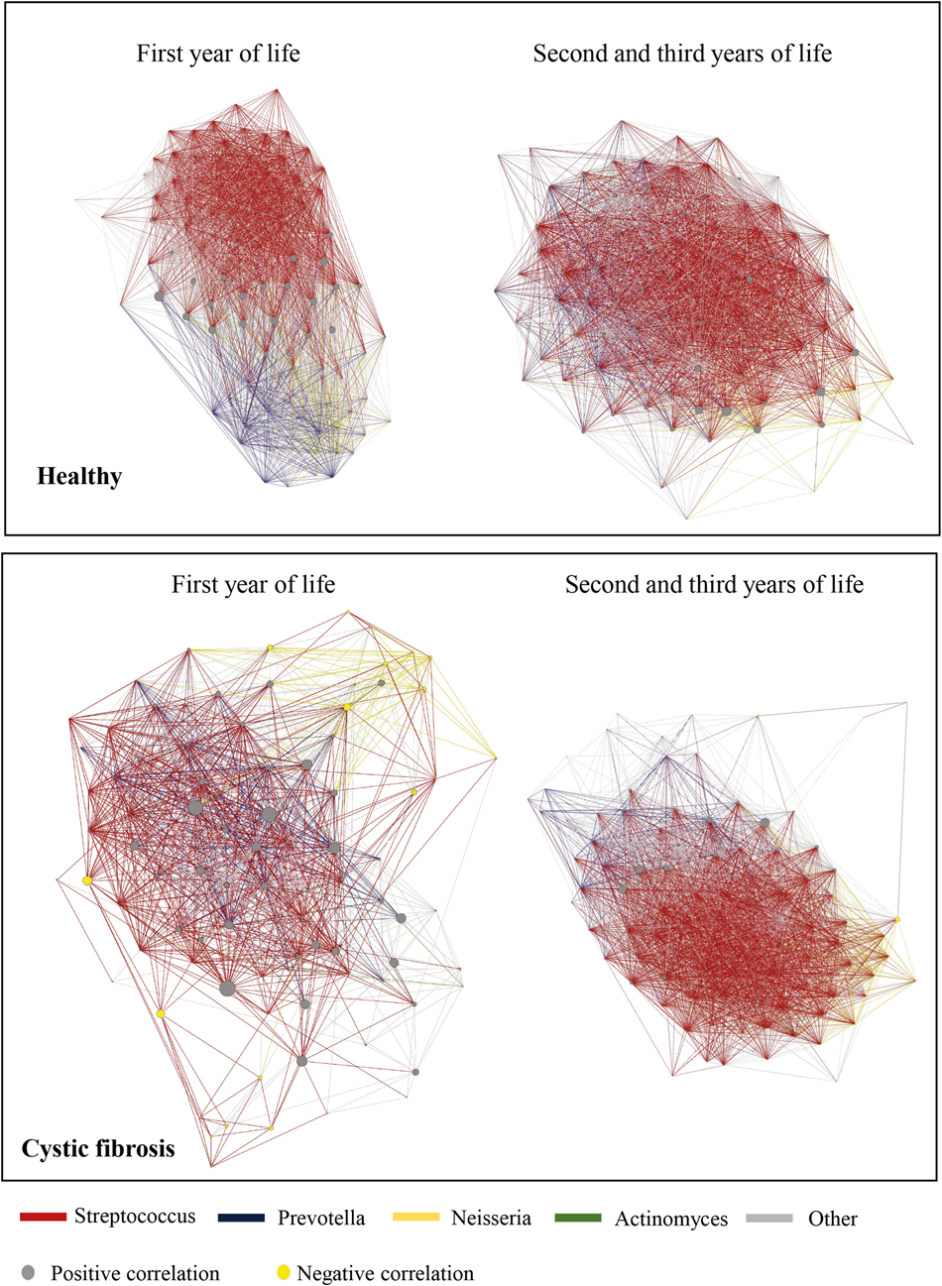
Ecological network analysis of species Spearman’s correlation matrices in healthy and diseased infants. The ForceAtlas algorithm was applied to Spearman’s rank correlation matrices, which were calculated from absolute species abundance tables of shotgun metagenomic sequencing data. Directed networks were generated by including only significant and strong positive correlations (*p* < 0.05, Spearman’s rank correlation coefficient > 0.60) and all significant negative correlations (*p* < 0.05), which are represented by grey and yellow nodes, respectively. Coloured edges visualise correlations that involve one of the four genera that explain most of the correlations (Streptococcus, Veillonella, Actinomyces and Neisseria), whereas all other edges are shown in grey. The size of network nodes refers to the corresponding betweenness centrality.

In the first years of life, strong positive species correlation networks were detected in healthy infants (Fig. 5). Negative species correlations were exclusively detected in CF infants. In healthy infants, degree centrality was significantly higher compared to age-matched CF infants but in both groups, degree centrality increased with age (Table 3). In healthy infants, closeness centrality was significantly higher than in age-matched CF infants (Table 3). Betweenness centrality was significantly lower in healthy compared to age-matched CF children. When comparing the three centrality network statistics between younger healthy infants and older CF infants, a significant difference was noted for betweenness centrality (Table 3).

**Table 3:** Centrality statistics of species co-occurrence network analysis for infants in the first year of life (A) and infants in the second and third year of life (B).

	Healthy A	Healthy B	CF A	CF B
Number of nodes	94	95	92	96
Number of edges	5506	6382	2456	5256
Number of negative correlations	0	0	56	12
	Closeness centrality	Betweenness centrality	Degree centrality	
Healthy A (median)	0.7	15.5	118.0	
Healthy B (median)	0.8	27.9	152.0	
Healthy A vs. Healthy B Mann–Whitney *p* value	0.0001	0.18	0.00003	
CF A (median)	0.5	68.1	47.0	
CF B (median)	0.7	34.3	112.0	
CF A vs. CF B Mann–Whitney *p* value	<0.00001	<0.00001	<0.00001	
Healthy A vs. CF A Mann–Whitney *p* value	<0.00001	<0.00001	<0.00001	
Healthy B vs. CF B Mann–Whitney *p* value	<0.00001	0.009	<0.00001	
Healthy A vs. CF B Mann–Whitney *p* value	0.27	0.03	0.73	

### An intermediate window of opportunity of close-to-healthy metagenomes in CF toddlers

After an initial postnatal period of instability characterised by negative species correlations and a more loosely organised species co-occurrence network, the CF microbial community structure stabilised during the following years two and three of age, and became similar to that of healthy infants. This intermediate period of an apparently healthy metagenome emerged in all CF infants irrespective of CFTR genotype, exocrine pancreatic status, anthropometry, lung function, the detection of *P. aeruginosa* (Supplementary Fig. 4 and Supplementary Table 4) or *S. aureus*-DNA (Supplementary Fig. 5).

### Stochastic detection of *P. aeruginosa*-DNA in healthy and CF airways

**Fig. 6:**
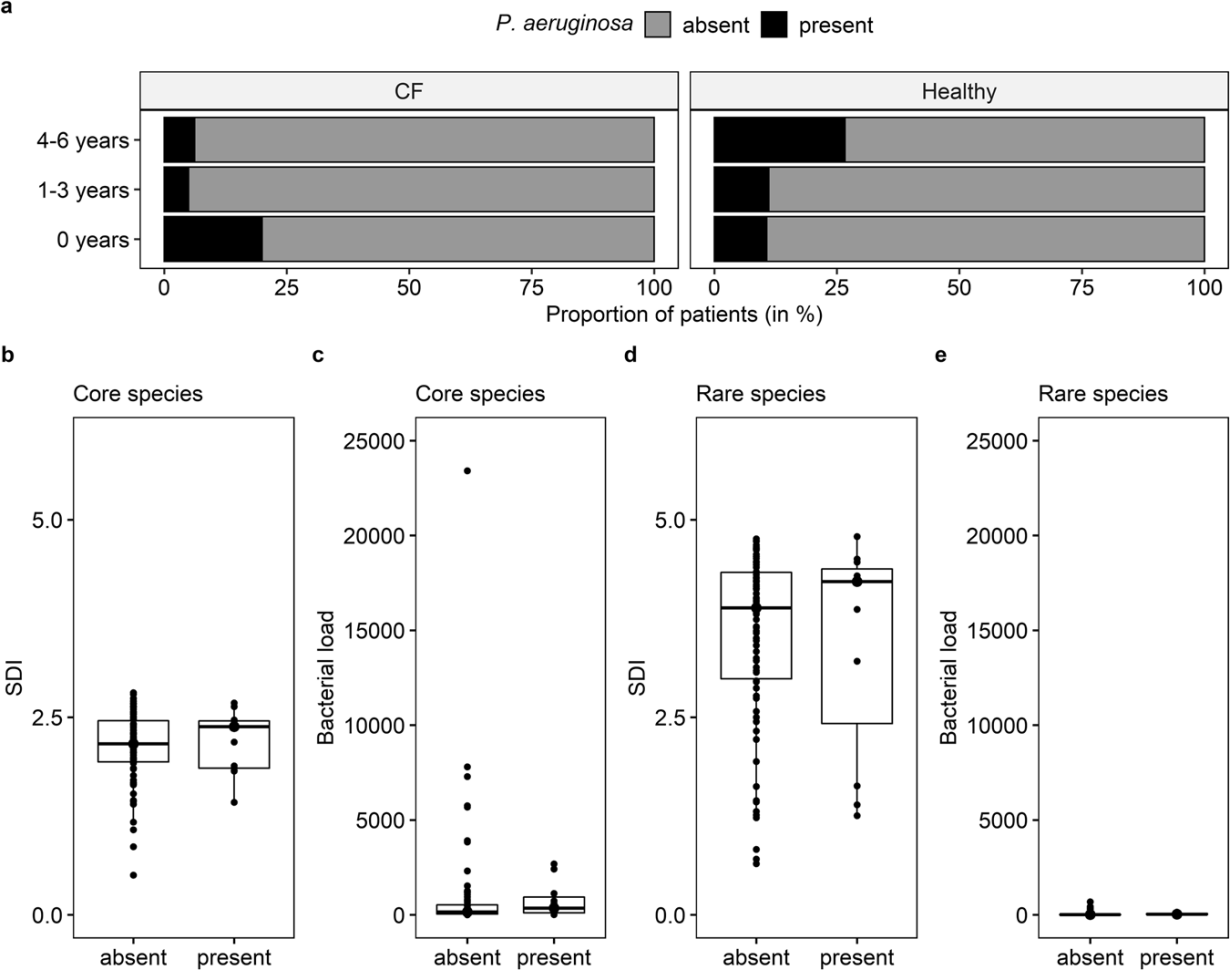
Impact of *P. aeruginosa*-DNA detection on the respiratory tract metagenome of CF and healthy infants. **a** Proportion of *P. aeruginosa*-DNA-positive and *P. aeruginosa*-DNA-negative children per age group and disease state. In the CF cohort, there were 5 infants below the age of 1, 20 between 1 and 3 years of age and 16 children between 4 and 6 years of age. In the healthy cohort, there were 28 infants below the age of 1, 9 children between 1 and 3 years of age and 15 preschool children were 4 and 6 years of age. **b**–**e** Shannon diversity indices (SDI) (**b**, **d**) and bacterial load of core (**c**) and rare species (**e**) based on the presence (right) and absence (left) of *P. aeruginosa*-DNA in cough swabs. The Mann–Whitney *U* test was applied to calculate significant differences between the two groups. No statistical differences were observed (*p* > 0.05). The centre line of the boxplot depicts the median (50th percentile). The lower and upper boundary of the box represent the first (25th percentile) and third (75th percentile) quartile, and hence define the interquartile range (IQR). Whiskers extend from the box to the largest/smallest non-outlier data point (1.5 × IQR).

Given the crucial effects of *P. aeruginosa* colonisation on the clinical course in CF lung disease, we performed detailed analysis of the detection of *P. aeruginosa*-DNA in healthy and CF children, and its impact on the respiratory microbial structure. In the first year of life, genetic material of *P. aeruginosa* was observed in healthy and CF infants, with the youngest *P. aeruginosa*-positive child being 3 weeks old (Fig. 6a). In that line, the detection of *P. aeruginosa*-DNA was not associated with disease state (Supplementary Fig. 6, left), i.e., both CF and healthy children showed similar detection rates of *P. aeruginosa*. Furthermore, neither age group (Supplementary Fig. 6, centre) nor season of sampling (Supplementary Fig. 6, right) were associated with *P. aeruginosa* detection. *P. aeruginosa* was always part of the 5% least abundant species. Detection of *P. aeruginosa*-DNA had neither a significant effect on diversity nor on absolute abundance of core and rare species (Fig. 6b–e). Longitudinally sampled CF infants received routine culture-dependent microbiological analyses as part of their clinical follow-ups, and we observed three different patterns of *P. aeruginosa*-DNA detection in this cohort: (1) the constant detection of *P. aeruginosa*-specific reads via metagenomics in culture-negative infants, (2) the complete absence of *P. aeruginosa*-specific reads via metagenomics and no culture-dependent detection of *P. aeruginosa* and (3) the absence of *P. aeruginosa*-specific reads via metagenomics until the child became culture-positive for the first time (Supplementary Fig. 7).

## Discussion

A range of CF respiratory tract microbiome studies has been published to investigate the microbial communities inhabiting the diseased respiratory tract in children^2–10^. These studies have applied partial 16 S 16S ribosomal RNA gene sequencing for taxonomic classification, which can lead to various taxonomic outcomes depending on the hypervariable region of amplification^39–41^. In this study, we applied deep shotgun metagenomic sequencing based on single-end reads of 75 bp length. For functional annotation it is currently recommended to generate long reads (>200 bp) and paired-end data, so that de novo assembly and subsequently alignment of the contigs against protein family databases is feasible^42,43^. We have consciously decided to forego the functional annotation because large numbers of protein families and bacterial genes remain annotated as ‘domain of unknown function’ and ‘hypothetical proteins’, respectively^44^. This has led to poor data reproducibility in past studies, especially when approaching different database versions^45^. However, our methodological approach enabled us to obtain quantitative information on core and rare bacteria, down to the species level by aligning reads against a curated reference database and by probing the maximum number of genome positions. In addition, we processed and sequenced negative controls, and included children with no history of pulmonary disease. This experimental set-up provided insights into the early community structure of the transient respiratory tract metagenome in health and CF. However, the major limitation of this study was the use of cough swabs to screen the respiratory tract metagenome. Whereas bronchoalveolar lavage (BAL) or induced sputum are gold standards for diagnosing lower airway infection, and investigating the lower respiratory tract metagenome^46,47^, throat swabs were often assigned as unsuitable for studying bacterial conditions in the CF lung due to a low test sensitivity (34–36%)^48,49^. While newborns and infants do not produce sputum, BAL is an invasive technique and inappropriate for use in longitudinal studies with short sampling intervals or for including a sufficient number of healthy controls. As a compromise, cough swabs were collected by trained CF paediatricians and sampling was accompanied by an obligate cough of the participant.

The healthy respiratory tract metagenome consists of core and rare species, which were here defined by the 95% of most abundant and the 5% of least abundant species, respectively. We found that the diversity of core species in the healthy microbial community developed gradually over a period of 4–6 years. Consequently, core species diversity was significantly higher in healthy preschool children (4–6 years of age) compared to healthy infants (first year of life). Interestingly, the diversity of rare species in a healthy microbial community was not found to change with age, suggesting that in the first year of life the rare species diversity has already been fully developed. However, a large interquartile range was apparent in each age group, indicating high variability of rare species diversity between the healthy infants in each age group.

It is also noteworthy that the healthy respiratory tract harboured more bacterial cells per human cells than the CF respiratory tract in all age groups. The increased absolute abundance of bacteria was not confined to specific species, but included all core and rare species equally. When microbial community profiles of children in terms of beta diversity were assessed, no disease-specific signature was recognisable in the early years of life. However, unsupervised learning algorithms revealed the patterns of a healthy microbial community profile (k3). The microbial community profiles in k3 were unique and completely distinct from members of the two other groups (k1–k2), but also from each other. The healthy cluster was characterised by increased bacterial loads of core and rare species and a higher diversity of rare species. The role of rare species colonising the human respiratory tract is still underestimated due to incomplete taxonomic databases and many metagenomic pipelines, which eliminate low-abundant taxa immediately^50–52^. The rare species community in the respiratory tract harbours more different species than the core respiratory tract community, and hence provides the microbial gene repertoire of the respiratory tract with tremendous functional flexibility^50^. Since increased diversity and bacterial load of rare species defined a unique and healthy microbial signature, the rare species of the respiratory tract could be subject for analysis in future studies.

Ecological network analysis is rarely applied to study species co-occurrence in the human host because the overall specificity of networks suffers when relative abundance data is used^26,28–30,53^. Since we were able to calculate absolute abundances of species from shotgun metagenomic sequencing data, robust co-occurrence network analysis was feasible. We found that degree centrality increased in the first years of life in healthy and CF infants, but there were always more connections per node in healthy than in age-matched CF infants. Ecologists commonly argue that the stability of a system increases as the number of links increases^54,55^. One could hence suggest that in terms of co-occurrence patterns, the healthy microbial community is more stable than the CF community and that stability increases with age. This suggestion is backed up by the discovery that in co-occurrence networks of healthy infants the closeness centrality was high, but betweenness centrality was low. If species in the healthy respiratory tract of infants get cleared by host defence mechanisms or go extinct, the linkage of the remaining positively correlating species in the respiratory tract is not affected and the single large cluster will be maintained. In CF infants, the exact opposite pattern was apparent. The number of bridging species was high, the number of central species was low. If bridging species get lost, there is a high risk of network fragmentation into independent clusters of co-occurrences, even in the absence of typical CF pathogens. In the second and third years of life, the number of bridging species in CF infants decreased, but remained high compared to age-matched healthy infants. In spite of permanent differences in betweenness centrality and with a significant time delay, we showed that the co-occurrence network of the early CF respiratory tract caught up and became similar to a healthy co-occurrence network over time.

While a personalised metagenome signature with many low-abundant and few dominant pulmonary pathogens has been described for adult CF patients and patients with end-stage lung disease^19–21^, we found no unique core microbial signature in the early respiratory tract of CF infants. On the contrary, in terms of relative species abundance and diversity of core and rare species, the CF metagenome was found to be highly similar to a healthy metagenome in the first 4 years of life. The main differences in the early years were the lower absolute abundance of bacteria and the prolonged instability of the microbial community after birth. Thereafter, an ~2-year period followed in which the CF airway microbial communities almost matched those of healthy infants. Then by ~4 years of age, the CF typical signature started to emerge. If this finding can be replicated in further geographically distant CF care settings^9,18,56,57^, we could develop an optimistic view that we have a time window^58^ of ~2 years to prevent the irreversible downhill course of the establishment of the CF typical airway metagenome. At the time of writing, the encouraging outcome of phase 3 clinical trials with CFTR modulators^59–61^ suggests that the early start of CFTR modulation may be the adequate preventive measure to acquire and retain a healthy airway metagenome in the CF airways.

*P. aeruginosa* is one of the hallmark pathogens of chronic airway infections in CF^22,23,62,63^. Losada and colleagues observed *P. aeruginosa*-DNA in all PI CF school children by DNA sequencing, even though some of the patients remained *P. aeruginosa*-negative in culture^19^. They suggested that *P. aeruginosa* acquisition may occur earlier than previously assumed but at low numbers. Deep shotgun metagenomic sequencing backed up by quality control measures now unravelled that not only CF, but also healthy infants come into contact with the environmental organism *P. aeruginosa* on a regular basis and from the very beginning. In the first year of life, trace amounts of *P. aeruginosa*-DNA were constantly tracked in a subgroup of CF and healthy infants. In some longitudinal CF samples, *P. aeruginosa*-specific DNA was always present, even though the samples remained *P. aeruginosa* negative in culture. Other longitudinal samples contained no *P. aeruginosa*-specific DNA until the pathogen grew in culture for the first time. It remains unknown whether different patterns of detection play a role in disease progression because the amount of *P. aeruginosa*-DNA was insufficient for estimating growth dynamics. Therefore, no statements can be made whether the pathogen was surviving at low numbers in the respiratory tract or whether residual DNA fragments were detected after elimination by host defences. Since the early eradication of *P. aeruginosa* is of importance to decrease morbidity and mortality in CF patients^22,23,62,63^, the scientific community strives to bring sensitive DNA sequencing tools into the clinic as diagnostic tool. Our novel finding of *P. aeruginosa*-DNA in a subset of healthy children emphasises the need to define detection thresholds and study their association with clinical course in future. Treatment is warranted when clinically relevant thresholds are exceeded. A further challenge is that the interpretation and quality of next-generation sequencing results depend on sample type, quality of sample collection, sampling device, method of sample processing, sequencing and the in silico pipeline, as well as the cleanliness of the laboratory and clinical environment^32–35,64,65^. As long as the scientific community does not agree on uniform practices and clinically relevant thresholds, quality-controlled culture-dependent diagnostics remains an irreplaceable tool to distinguish the regular encounter of children with the environmental organism from harmful *P. aeruginosa* airway colonisation, which requires immediate medical intervention.

In conclusion, deep shotgun metagenomic sequencing of carefully collected cough swabs of CF and healthy children at a very early age provided unprecedented insights into the early community structure of the transient respiratory tract metagenome in health and CF. It became evident that in the first years of life, the CF and healthy microbial community structures are similar. Based on the diversity of core species, a significant difference was apparent only by the age of 4 years. In terms of beta diversity, no CF-specific signature was apparent. We could associate a healthy microbial community signature with increased bacterial loads of core and rare species, a higher diversity of the rare species, a strong positive species correlation network with high degree centrality, high closeness centrality and low betweenness centrality, and the complete absence of negative species correlations. Species co-occurrence patterns in CF infants were defined by the presence of negative species correlations, high betweenness centrality but low degree and closeness centrality. The CF correlation network in the early years of life was assumed to be prone to network fragmentation due to the high number of bridging species.

The presence of low-abundant *P. aeruginosa*-DNA did neither influence alpha nor beta diversity metrics and was not disease associated. Children seem to come into contact with the environmental organism *P. aeruginosa* on a regular basis without requiring medical intervention. It is hence critical to agree on detection thresholds to distinguish medical relevant pathogens from harmless background patterns. Until then, culture-dependent microbiology remains an irreplaceable tool in CF clinical microbiology.

## Methods

### Participants

Fifty-two deep cough swabs from healthy children between 0 and 6 years of age, with no medical history or suspicion of pulmonary diseases were collected by trained paediatricians, during the regular preventive medical examination or at kindergartens and local parent–child meetings in Hannover, Germany. Forty-one children with CF were recruited from the Cystic Fibrosis Outpatient Clinic at Hannover Medical School (MHH), Germany. Eleven CF participants were screened longitudinally after diagnosis following the CF newborn screening (Table 1 and Supplementary Table 1). All CF participants were regularly seen and monitored by CF specialists at the MHH since the age of diagnosis. The clinical study was approved by the ethics committee of MHH (No. 7674). The parents or legal guardians gave written consent prior to sample collection.

### Sample collection

Deep cough swabs were collected with sterile cotton swabs (6.0 × 6.0 mm) from specialised CF paediatricians. Sampling was accompanied by an obligate cough of the participant. Swabs were placed directly into DNA LoBind Tubes (Eppendorf, #022431021); tips facing downwards. Swab handles were cut with sterile scissors and samples were immediately stored at −80 °C until further processed. The latent period between sample collection and quick freezing was 15–30 s.

### Preparation of a clean environment

Since the type and extent of contamination varies between laboratory environments, and within one laboratory over time^31–33^, we established a standard cleaning procedure of the laboratory environment which was uniformly conducted before sample processing. Before sample processing, aliquots for use in all biological samples and negative controls were prepared simultaneously in a UV PCR workstation, then sealed with Parafilm and stored as required, so that all samples were treated with the same kits by lot number. The day before sample processing, the workstation and laboratory equipment were cleaned with 5% sodium hypochlorite solution (w/v) and left for overnight exposure. During preparation and sample processing in the UV PCR workstation, disposable laboratory coats, sterile gloves, mouth and hair protection were worn. Negative controls (blank swabs and empty water controls) were stored, processed and sequenced in parallel with patient samples for a constant quality control of experiments.

### DNA extraction and fragmentation

Cotton swabs were soaked in TE buffer (200 µl, 0.1×), placed in a dry-ice-absolute-ethanol mixture for 4 min and then in a heating block (65 °C, 3 min). Freezing–heating cycles were repeated three times. The tubes were sealed with Parafilm and loaded onto the S220 Focused-ultrasonicator (Covaris, programme 1, Supplementary Table 5). Sterile syringes were used for pricking holes into the bottom of sterile 0.5-ml Eppendorf tubes, which were then stacked on top of 1.5-ml Eppendorf tubes. Solution and swab tip were transferred into the manipulated 0.5-ml Eppendorf tube. A quick spin was performed (30 s) and the 0.5-ml Eppendorf tubes were discarded. The flow-through solution (130 µl) was pipetted in a Covaris microTUBE. The tube was sealed with Parafilm. The Covaris (programme 2, Supplementary Table 5) was started, yielding DNA fragments of 200 bp length. The solution was centrifuged (3 min, 13,200 × *g*, 25 °C). The supernatant (130 µl) was mixed with AMPure XP Beads (156 µl) and incubated (25 °C, 5 min). The tube was placed on a magnetic rack. The clear supernatant was discarded and the pellet was washed with ethanol (80%) three times. The pellet was resuspended in TE buffer (30 µl, 0.1×). The solution was incubated (25 °C, 2 min) and placed on the magnetic rack. The clear solution was pipetted in a PCR tube for library preparation.

### Library preparation and DNA sequencing

The protocol for use with NEBNext Ultra II DNA Library Prep Kit for Illumina (E7645, E7103) was followed without size selection, with NEBNext unique dual index primer pairs and a maximum number of 12 PCR cycles. The Illumina NextSeq 500/550 platform was used for sequencing (High Output Kit v2.5, 75 cycles, single-end reads, #20024906). The flow cell was under clustered (1.3 pM instead of default 1.5 pM) to prevent cluster overlaps.

### Taxonomic classification

The whole metagenomic sequencing alignment pipeline version 1.1 of Davenport and Scheithauer^66^ was employed for taxonomic classification with default adjustments. An in-house reference database (see ‘Data availability’ section) was created for the alignment process with complete reference genomes of bacteria (*n* = 2598), DNA viruses (*n* = 38) and human chromosomes (*n* = 23), which were extracted from the NCBI RefSeq database. Raw microbial reads were normalised to human reads as described by Losada et al.^19^. The 95% of most abundant bacterial species (core species) and the 5% of least abundant species (rare species) were obtained separately from CF and healthy samples. The detection of core species in the respiratory tract was verified by the *k*-mer and marker gene-based tools Centrifuge^67^ and Metaphlan2^51^, respectively.

### Statistical analysis

For comparing two independent groups and more than two groups, the non-parametric Mann–Whitney *U* test and the Kruskal–Wallis rank test were applied, respectively. For two groups, the effect size *r* was calculated, which is the Mann–Whitney *U* test statistics divided by the square-rooted sample size. For more than two groups, the epsilon-squared effect size (e2) was obtained. Confidence intervals (CI) were identified. The Conover–Iman test with Benjamini–Hochberg adjustment^68^ was used for multiple comparisons between group levels. Fisher’s exact test was employed for statistical evaluation of count data with small sample sizes. For hierarchical clustering (Ward’s method), the dataset’s clustering tendency was evaluated with the Hopkins statistic^36^ and a Euclidean distance matrix was built. Bray–Curtis dissimilarity indices were obtained for nmds^69^ (without autotransform adjustment, *k* = 3, stress = 0.07). A permutation test (envfit^69^, permutations = 1000) was used to establish relationships between the nmds plot and metadata variables. For predicting the presence or absence of *P. aeruginosa* in samples with low numbers of reads (<1× coverage), the tool raspir^70^ was approached to study the read distribution across the bacterial genome. R statistical software was used for data analyses, including the vegan package^69^ for community ecology analysis and the rcompanion package for statistical testing^71^. All the scripts and input tables are publicly available (see ‘Code availability’ section). For ecological network analysis, the best practice guidelines for co-occurrence network construction were followed^53^. Spearman’s rank correlation matrices were generated from absolute abundance tables of the 99% most abundant species and correlations with *p* values < 0.05 were extracted. For positive correlations, only strong correlations were included (Spearman’s rank correlation coefficient > 0.60). The open-source software Gephi^72^ (https://gephi.org/) was utilised for directed network analyses with the continuous graph layout algorithm ForceAtlas^38^ (inertia = 0.1, repulsion = 10,000.0, attraction = 10.0, maximum = 10.0, auto stabilisation = TRUE, gravity = 30.0). The network parameters degree centrality, closeness centrality and betweenness centrality were obtained. Degree centrality measures the numbers of connections of a node. Closeness centrality calculates the shortest distance of a node to all other nodes in the network^37^, where a high value refers to a more central node. Betweenness centrality measures how often a node is bridged by the shortest pathway of two other nodes^37^.

### Reporting summary

Further information on research design is available in the Nature Research Reporting Summary linked to this article.

## Supplementary information

Supplementary Information

Reporting Summary

## Data Availability

The microbial sequencing data are stored in the European Nucleotide Archive (study accession number PRJEB38221). The reference database, R scripts and input files (absolute abundance estimations of species per sample, metadata) are available from https://github.com/mmpust/airway-metagenome-infants.

## References

[1] Dickson RP, Erb-Downward JR, Martinez FJ, Huffnagle GB (2016). The microbiome and the respiratory tract. Annu. Rev. Physiol..

[2] Frayman KB (2019). Differences in the lower airway microbiota of infants with and without cystic fibrosis. J. Cyst. Fibros..

[3] Frayman KB (2017). The lower airway microbiota in early cystic fibrosis lung disease: a longitudinal analysis. Thorax.

[4] Ahmed B (2019). Longitudinal development of the airway microbiota in infants with cystic fibrosis. Sci. Rep..

[5] Laguna TA (2016). Airway microbiota in bronchoalveolar lavage fluid from clinically well infants with cystic fibrosis. PLoS ONE.

[6] Zemanick ET (2017). Airway microbiota across age and disease spectrum in cystic fibrosis. Eur. Respir. J..

[7] Coburn B (2015). Lung microbiota across age and disease stage in cystic fibrosis. Sci. Rep..

[8] Madan JC (2012). Serial analysis of the gut and respiratory microbiome in cystic fibrosis in infancy: interaction between intestinal and respiratory tracts and impact of nutritional exposures. MBio.

[9] Muhlebach MS (2018). Initial acquisition and succession of the cystic fibrosis lung microbiome is associated with disease progression in infants and preschool children. PLoS Pathog..

[10] Kirst ME, Baker D, Li E, Abu-Hasan M, Wang GP (2019). Upper versus lower airway microbiome and metagenome in children with cystic fibrosis and their correlation with lung inflammation. PLoS ONE.

[11] Man WH, De Steenhuijsen Piters WAA, Bogaert D (2017). The microbiota of the respiratory tract: gatekeeper to respiratory health. Nat. Rev. Microbiol..

[12] Wypych TP, Wickramasinghe LC, Marsland BJ (2019). The influence of the microbiome on respiratory health. Nat. Immunol..

[13] Bassis CM (2015). Analysis of the upper respiratory tract microbiotas as the source of the lung and gastric microbiotas in healthy individuals. MBio.

[14] Dickson RP (2015). Spatial variation in the healthy human lung microbiome and the adapted island model of lung biogeography. Ann. Am. Thorac. Soc..

[15] Lyczak JB, Cannon CL, Pier GB (2002). Lung infections associated with cystic fibrosis. Clin. Microbiol. Rev..

[16] Henry RL, Mellis CM, Petrovic L (1992). Mucoid *Pseudomonas aeruginosa* is a marker of poor survival in cystic fibrosis. Pediatr. Pulmonol..

[17] Kosorok MR (2001). Acceleration of lung disease in children with cystic fibrosis after *Pseudomonas aeruginosa* acquisition. Pediatr. Pulmonol..

[18] Cuthbertson L (2020). Lung function and microbiota diversity in cystic fibrosis. Microbiome.

[19] Losada PM (2016). The cystic fibrosis lower airways microbial metagenome. ERJ Open Res..

[20] Price KE (2013). Unique microbial communities persist in individual cystic fibrosis patients throughout a clinical exacerbation. Microbiome.

[21] Surette MG (2014). The cystic fibrosis lung microbiome. Ann. Am. Thorac. Soc..

[22] Schelstraete P, Haerynck F, Van daele S, Deseyne S, De Baets F (2013). Eradication therapy for *Pseudomonas aeruginosa* colonization episodes in cystic fibrosis patients not chronically colonized by *P. aeruginosa*. J. Cyst. Fibros..

[23] Rosenfeld M (2010). Baseline characteristics and factors associated with nutritional and pulmonary status at enrollment in the cystic fibrosis EPIC observational cohort. Pediatr. Pulmonol..

[24] Barlow JT, Bogatyrev SR, Ismagilov RF (2020). A quantitative sequencing framework for absolute abundance measurements of mucosal and lumenal microbial communities. Nat. Commun..

[25] Harrison, J. G., Calder, W. J., Shuman, B. & Buerkle, C. A. The quest for absolute abundance: the use of internal standards for DNA-based community ecology. *Mol. Ecol. Resour.*10.1111/1755-0998.13247 (2020). 10.1111/1755-0998.13247 32889760

[26] Knight R (2018). Best practices for analysing microbiomes. Nat. Rev. Microbiol..

[27] Morton JT (2019). Establishing microbial composition measurement standards with reference frames. Nat. Commun..

[28] Aitchison J (1982). The statistical analysis of compositional data. J. R. Stat. Soc. Ser. B.

[29] Gloor GB, Macklaim JM, Vu M, Fernandes AD (2016). Compositional uncertainty should not be ignored in high-throughput sequencing data analysis. Austrian J. Stat..

[30] Gloor GB, Macklaim JM, Pawlowsky-Glahn V, Egozcue JJ (2017). Microbiome datasets are compositional: and this is not optional. Front. Microbiol..

[31] Glassing A, Dowd SE, Galandiuk S, Davis B, Chiodini RJ (2016). Inherent bacterial DNA contamination of extraction and sequencing reagents may affect interpretation of microbiota in low bacterial biomass samples. Gut Pathog..

[32] Weiss S (2014). Tracking down the sources of experimental contamination in microbiome studies. Genome Biol..

[33] Weyrich LS (2019). Laboratory contamination over time during low‐biomass sample analysis. Mol. Ecol. Resour..

[34] Salter SJ (2014). Reagent and laboratory contamination can critically impact sequence-based microbiome analyses. BMC Biol..

[35] Eisenhofer R (2019). Contamination in low microbial biomass microbiome studies: Issues and recommendations. Trends Microbiol..

[36] Hopkins B, Skellam JG (1954). A new method for determining the type of distribution of plant individuals. Ann. Bot..

[37] Golbeck, J. *Analyzing the Social Web: Network Structure and Measures* (Elsevier, Burlington, VA, 2013).

[38] Jacomy M, Venturini T, Heymann S, Bastian M (2014). ForceAtlas2, a continuous graph layout algorithm for handy network visualization designed for the Gephi software. PLoS ONE.

[39] Bukin YS (2019). The effect of 16s rRNA region choice on bacterial community metabarcoding results. Sci. Data.

[40] Kim M, Morrison M, Yu Z (2011). Evaluation of different partial 16S rRNA gene sequence regions for phylogenetic analysis of microbiomes. J. Microbiol. Methods.

[41] Yang B, Wang Y, Qian PY (2016). Sensitivity and correlation of hypervariable regions in 16S rRNA genes in phylogenetic analysis. BMC Bioinformatics.

[42] Tamames J, Cobo-Simón M, Puente-Sánchez F (2019). Assessing the performance of different approaches for functional and taxonomic annotation of metagenomes. BMC Genomics.

[43] Góngora-Castillo E, Buell CR (2013). Bioinformatics challenges in de novo transcriptome assembly using short read sequences in the absence of a reference genome sequence. Nat. Prod. Rep..

[44] Zhang X (2016). Assignment of function to a domain of unknown function: DUF1537 is a new kinase family in catabolic pathways for acid sugars. Proc. Natl Acad. Sci. USA.

[45] Tomczak A (2018). Interpretation of biological experiments changes with evolution of the Gene Ontology and its annotations. Sci. Rep..

[46] Blau H (2014). Induced sputum compared to bronchoalveolar lavage in young, non-expectorating cystic fibrosis children. J. Cyst. Fibros..

[47] Eyns H (2018). Respiratory bacterial culture sampling in expectorating and non-expectorating patients with cystic fibrosis. Front. Pediatr..

[48] Jung A (2002). Sequential genotyping of *Pseudomonas aeruginosa* from upper and lower airways of cystic fibrosis patients. Eur. Respir. J..

[49] Equi AC, Pike SE, Davies J, Bush A (2001). Use of cough swabs in a cystic fibrosis clinic. Arch. Dis. Child.

[50] Jousset A (2017). Where less may be more: how the rare biosphere pulls ecosystems strings. ISME J..

[51] Truong DT (2015). MetaPhlAn2 for enhanced metagenomic taxonomic profiling. Nat. Methods.

[52] Huson DH, Auch AF, Qi J, Schuster SC (2007). MEGAN analysis of metagenomic data. Genome Res..

[53] Berry D, Widder S (2014). Deciphering microbial interactions and detecting keystone species with co-occurrence networks. Front. Microbiol..

[54] Paine RT (1969). A note on trophic complexity and community stability. Am. Nat..

[55] MacArthur R (1955). Fluctuations of animal populations and a measure of community stability. Ecology.

[56] Esther CR (2019). Mucus accumulation in the lungs precedes structural changes and infection in children with cystic fibrosis. Sci. Transl. Med..

[57] Pittman JE (2017). Association of antibiotics, airway microbiome, and inflammation in infants with cystic fibrosis. Ann. Am. Thorac. Soc..

[58] Hampton TH (2014). The microbiome in pediatric cystic fibrosis patients: the role of shared environment suggests a window of intervention. Microbiome.

[59] Heijerman HGM (2019). Efficacy and safety of the elexacaftor plus tezacaftor plus ivacaftor combination regimen in people with cystic fibrosis homozygous for the F508del mutation: a double-blind, randomised, phase 3 trial. Lancet.

[60] Middleton PG (2019). Elexacaftor-tezacaftor-ivacaftor for cystic fibrosis with a single Phe508del allele. N. Engl. J. Med..

[61] Bell SC (2020). The future of cystic fibrosis care: a global perspective. Lancet Respir. Med..

[62] Emerson J, Rosenfeld M, McNamara S, Ramsey B, Gibson RL (2002). *Pseudomonas aeruginosa* and other predictors of mortality and morbidity in young children with cystic fibrosis. Pediatr. Pulmonol..

[63] Ratjen F, Munck A, Kho P, Angyalosi G (2010). Treatment of early *Pseudomonas aeruginosa* infection in patients with cystic fibrosis: the ELITE trial. Thorax.

[64] Amrane S, Lagier JC (2018). Metagenomic and clinical microbiology. Hum. Microbiome J..

[65] Jorth P (2019). Direct lung sampling indicates that established pathogens dominate early infections in children with cystic fibrosis. Cell Rep..

[66] Davenport, C. & Scheithauer, T. Wochenende - a whole genome/metagenome sequencing alignment pipeline (version 1.1). Github repository, https://github.com/MHH-RCUG/Wochenende (2017).

[67] Kim D, Song L, Breitwieser FP, Salzberg SL (2016). Centrifuge: rapid and sensitive classification of metagenomic sequences. Genome Res.

[68] Pounds S, Cheng C (2006). Robust estimation of the false discovery rate. Bioinformatics.

[69] Dixon P (2003). VEGAN, a package of R functions for community ecology. J. Veg. Sci..

[70] Pust, M. M. Rare species identifier for whole shotgun metagenomics experiments. Github repository, https://github.com/mmpust/raspir (2020).

[71] Mangiafico, S. *An R Companion for the Handbook of Biological Statistics* https://rcompanion.org/rcompanion/ (2016).

[72] Bastian, M., Heymann, S., Jacomy, M. Gephi: an open source software for exploring and manipulating networks. In *Proc. Third International ICWSM Conference on Weblogs and Social Media*, 361–362 (ICWSM, San Jose, California, USA, 2009).

