# Supplementary material for: The human respiratory tract microbial community structures in healthy and cystic fibrosis infants
Source: NPJ Biofilms Microbiomes. 2020 Dec 15;6:61. doi: 10.1038/s41522-020-00171-7 (PMC7738502; doi:10.1038/s41522-020-00171-7)
Supplement: Supplementary file 1 — Supplementary Information [file 41522_2020_171_MOESM1_ESM.pdf]

The human respiratory tract microbial community structures in healthy and cystic fibrosis infants

Marie-Madlen Pust[1,2], Lutz Wiehlmann[3], Colin Davenport[3], Isa Rudolf[1,2], Anna-Maria Dittrich[1,2] and Burkhard Tümmler[1,2]

[1]Clinic for Paediatric Pneumology, Allergology, and Neonatology, Hannover Medical School, Germany

[2] Biomedical Research in Endstage and Obstructive Lung Disease Hannover (BREATH), German Center for Lung Research, Hannover Medical School, Germany

[3]Research Core Unit Genomics, Hannover Medical School, Germany

Corresponding author: tuemmler.burkhard@mh-hannover.de

Supplementary Material

Supplementary Figures

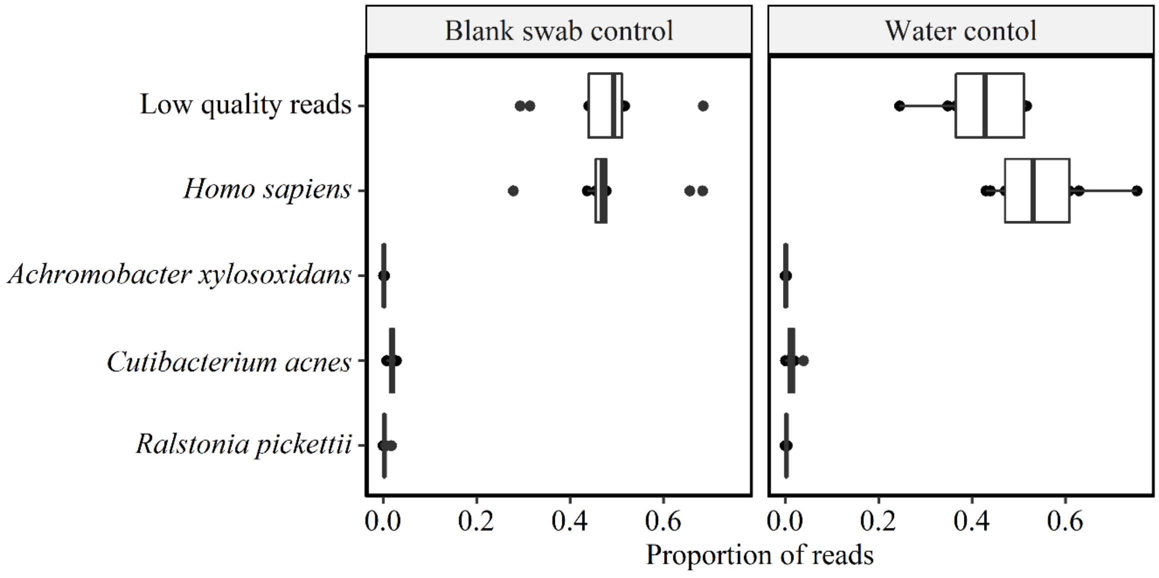

Supplementary Figure 1. Experimental controls reveal high-abundance of low quality and human sequence reads and typical patterns of microorganisms. Blank swabs (**left**) and empty water controls (**right**) were prepared and sequenced in parallel with patient samples. Raw counts were divided by sequencing depth (proportion of reads). Most of the reads from experimental controls aligned to the human reference genome or were discarded as low-quality reads. Three microorganisms were constantly tracked in all experimental controls, including *Cutibacterium acnes*, *Ralstonia pickettii* and *Achromobacter xylosoxidans*. The background contamination of independent sequencing runs was reduced to a minimum by implementing the ultra-clean guidelines that have been proposed in the method section. The lower and upper boundary of the boxplot represent the first (25th percentile) and third (75th percentile) quartile and hence define the interquartile range (IQR). Whiskers extend from the box to the largest/smallest non-outlier data point (1.5 * IQR).

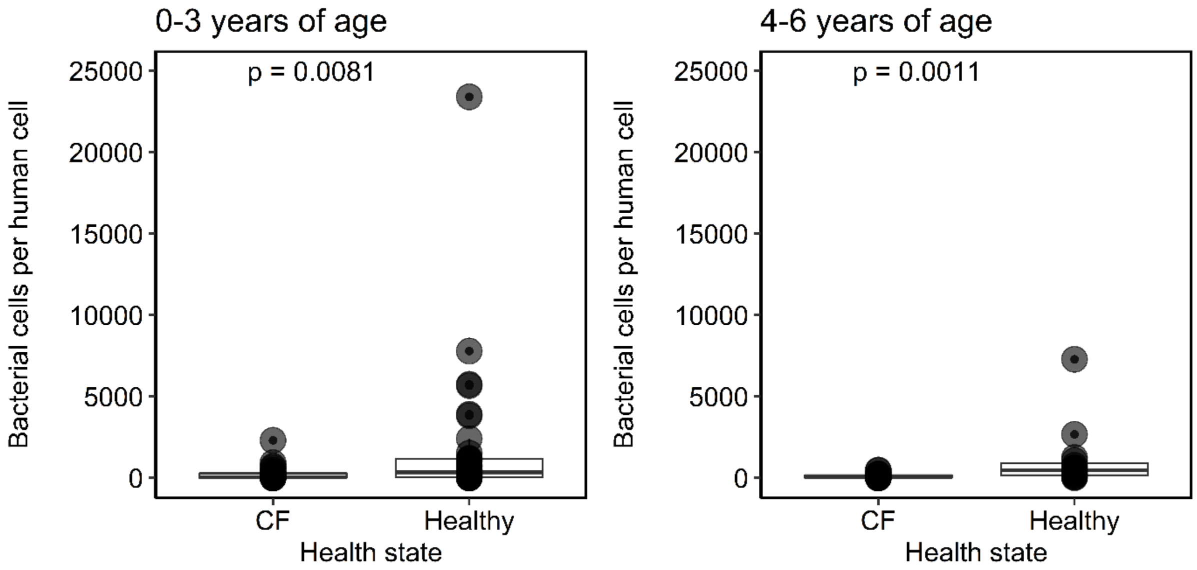

Supplementary Figure 2. Bacterial load in the respiratory tract of healthy and CF children. (**Left**) Comparison of bacterial cells per human cell between healthy and CF infants in the first three years of life. Healthy children seemed to have more bacterial cells per human cell than CF children (Wilcoxon p-value = 0.008, r = 0.34, CI = 0.10 – 0.55). (**Right**) Comparison of bacterial cells per human cell in healthy and CF pre-school children between four and six years of age. Healthy children showed higher bacterial loads than CF children (Wilcoxon p-value = 0.001, r = 0.57, CI = 0.28 – 0.77). The lower and upper boundary of the boxplot represent the first (25th percentile) and third (75th percentile) quartile and hence define the interquartile range (IQR). Whiskers extend from the box to the largest/smallest non-outlier data point (1.5 * IQR).

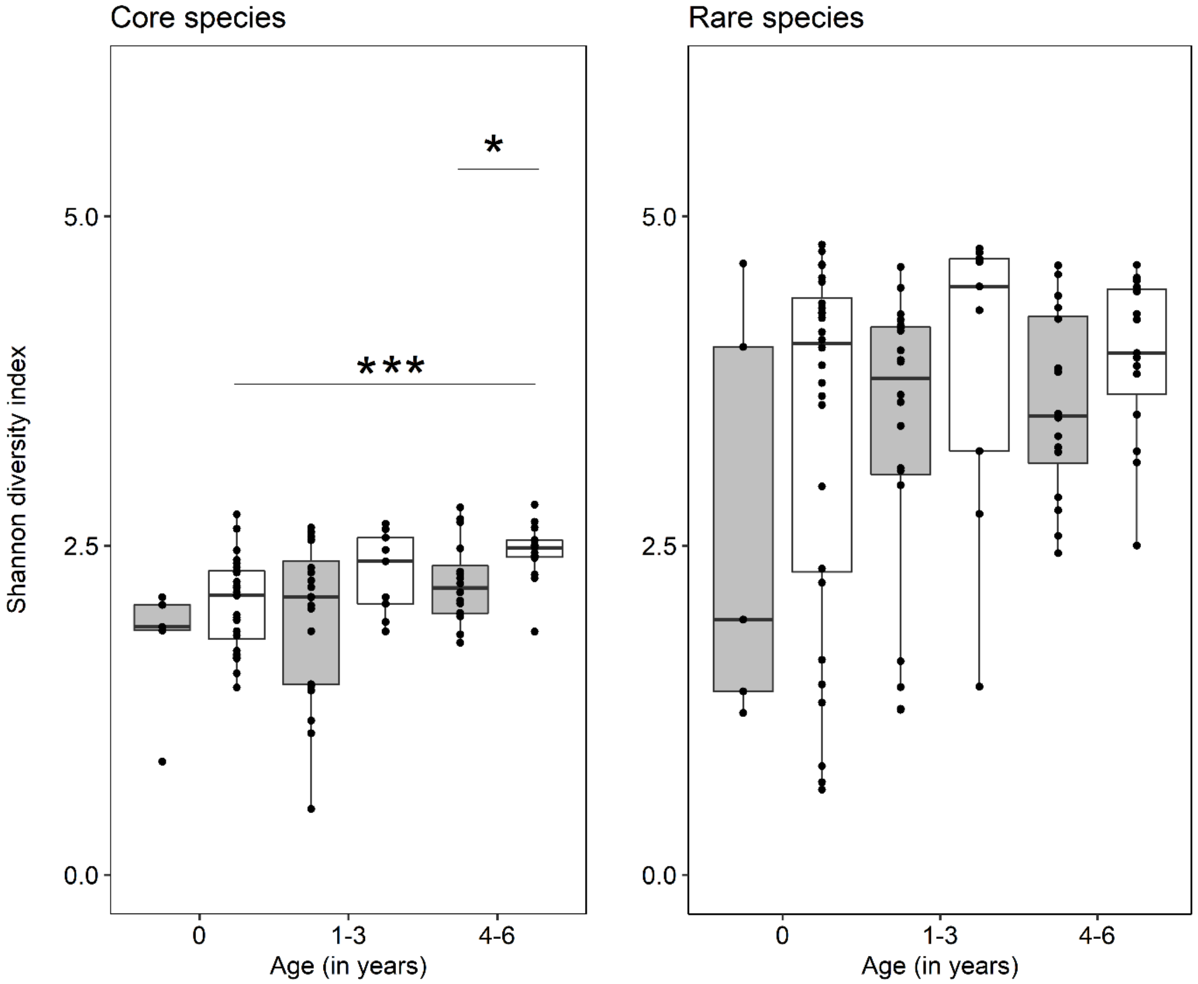

Supplementary Figure 3. Comparison of Shannon diversity indices between CF children (grey) and healthy children (white) in different age groups and age-dependent changes in the healthy and CF groups. (**Left**) Between the age of four and six years, the diversity of the 95% most abundant species was significantly different between healthy and CF children (Wilcoxon rank-sum p-value = 0.02, r = 0.41, CI = 0.08 – 0.71). In healthy children, the species diversity of core species constantly increased over time (Kruskal-Wallis p-value = 0.0003, e2 = 0.28, CI = 0.11 – 0.52). In CF children, no significant difference in diversity of core species was observed with increasing age (Kruskal-Wallis p-value > 0.05, e2 = 0.09, CI = 0.01 – 0.29). (**Right**) Concerning the rare taxa, defined as the 5% least abundant species, significant differences between CF and healthy children were not observed. Pairwise comparisons were done using the Conover-Iman test with Benjamini-Hochberg adjustment

(pairwise p-values are given in the diagram with * = $p < 0.05$, *** = $p < 0.001$). The lower and upper boundary of the boxplot represent the first (25th percentile) and third (75th percentile) quartile and hence define the interquartile range (IQR). Whiskers extend from the box to the largest/smallest non-outlier data point (1.5 * IQR).

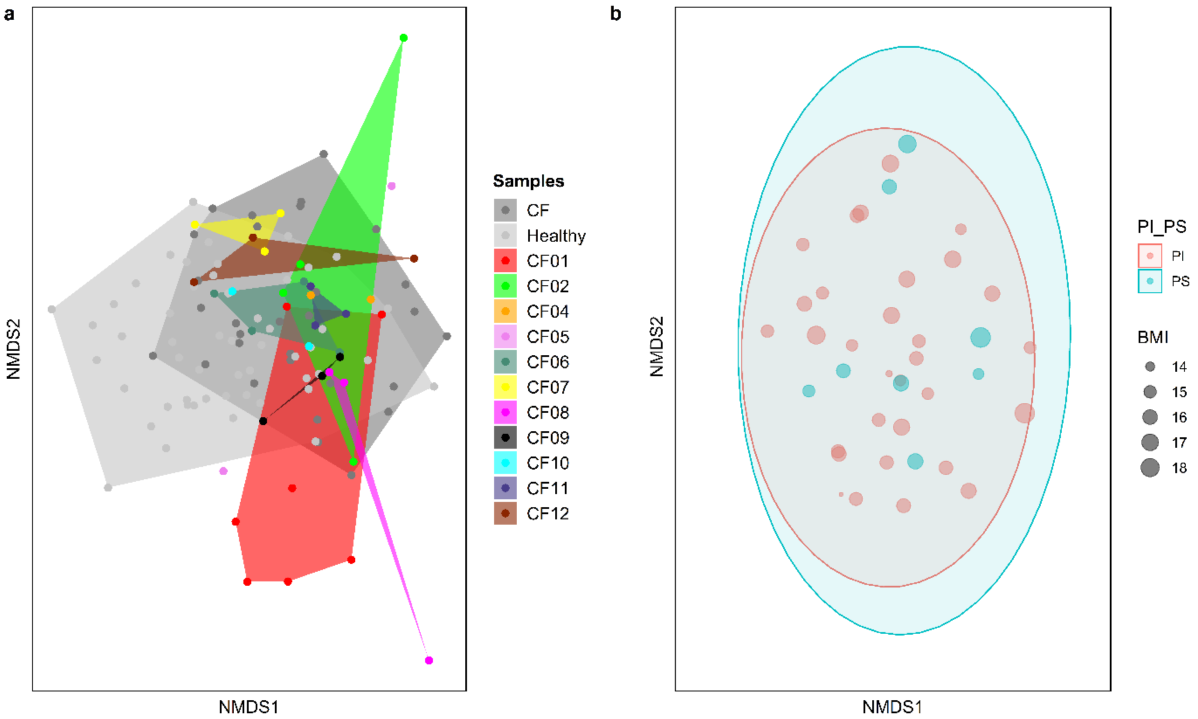

Supplementary Figure 4. Non-metric multidimensional scaling of Bray-Curtis dissimilarity indices of the CF cohort (a) Investigation of longitudinal samples. Each colour represents one longitudinally sampled CF infant. No individual CF signature was observed. The longitudinal samples of one patient clustered across the spectrum of healthy and CF children. **(b)** Investigations of cross-sectional CF microbial community profiles revealed extensive overlap between pancreatic insufficient (PI) and pancreatic sufficient (PS) patients. A permutation test was approached to fit clinical metadata (Supplementary Table 4) onto the ordination. None of the variables explained cluster behaviour.

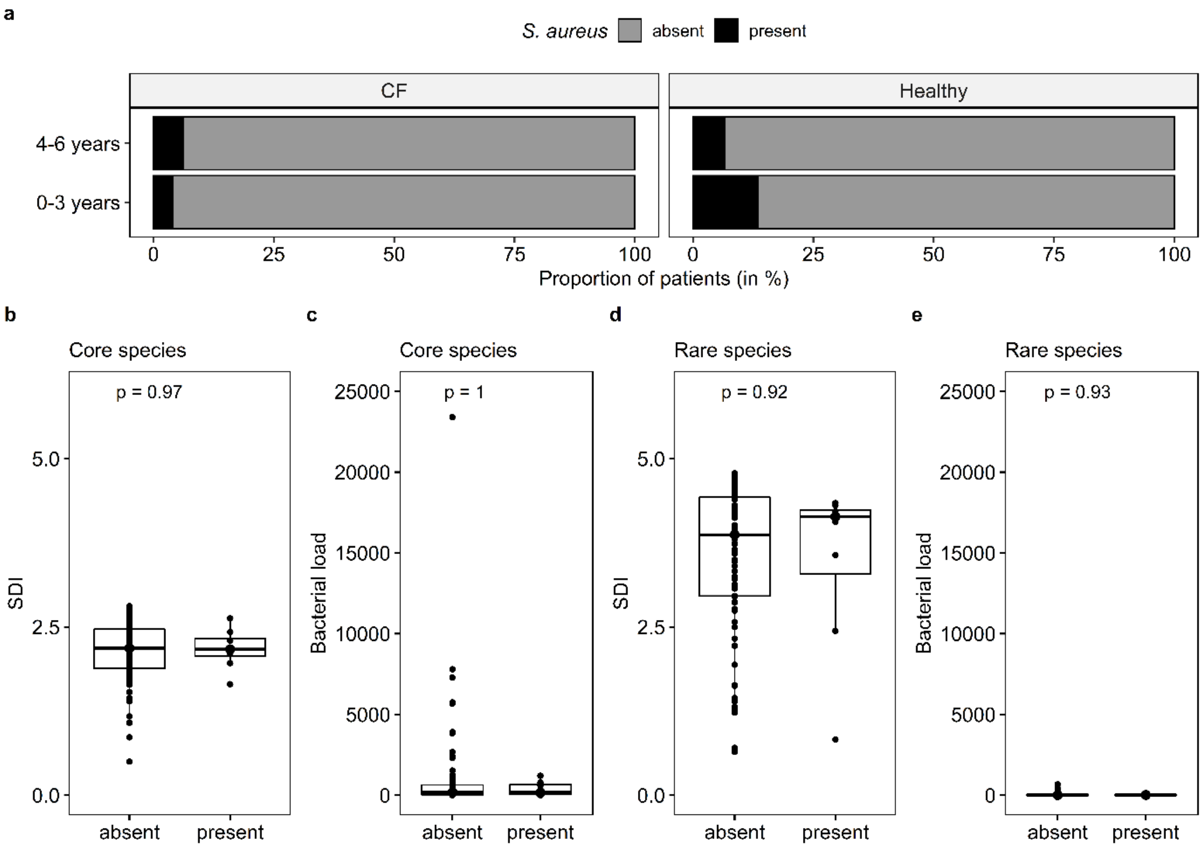

Supplementary Figure 5. Impact of *Staphylococcus aureus* - DNA detection on the respiratory tract metagenome of CF and healthy infants. (a) Proportion of *S. aureus* – positive children in the CF and healthy cohorts. In the CF cohort, there were five infants below the age of one, twenty between one to three years of age and sixteen children between four to six years of age. In the healthy cohort, there were twenty-eight infants below the age of one, nine children between one to three years of age and fifteen pre-school children were four to six years of age. The presence of *S. aureus* as a rare species in the respiratory tract did not influence **(b)** core species diversity (SDI, Wilcoxon p- value = 0.97, r = 0, CI = -0.2 – 0.2), **(c)** absolute abundance of core species (Wilcoxon p-value = 1, r = 0, CI = -0.2 – 0.2), **(d)** rare species diversity (Wilcoxon p-value = 0.92, r = 0.006, CI = -0.2 – 0.2) and **(e)** absolute abundance of rare species (Wilcoxon p-value = 0.93, r = 0.01, CI = -0.2 – 0.2). The lower and upper boundary of the boxplot represent the first (25th percentile) and third (75th percentile)

quartile and hence define the interquartile range (IQR). Whiskers extend from the box to the largest/smallest non-outlier data point (1.5 * IQR).

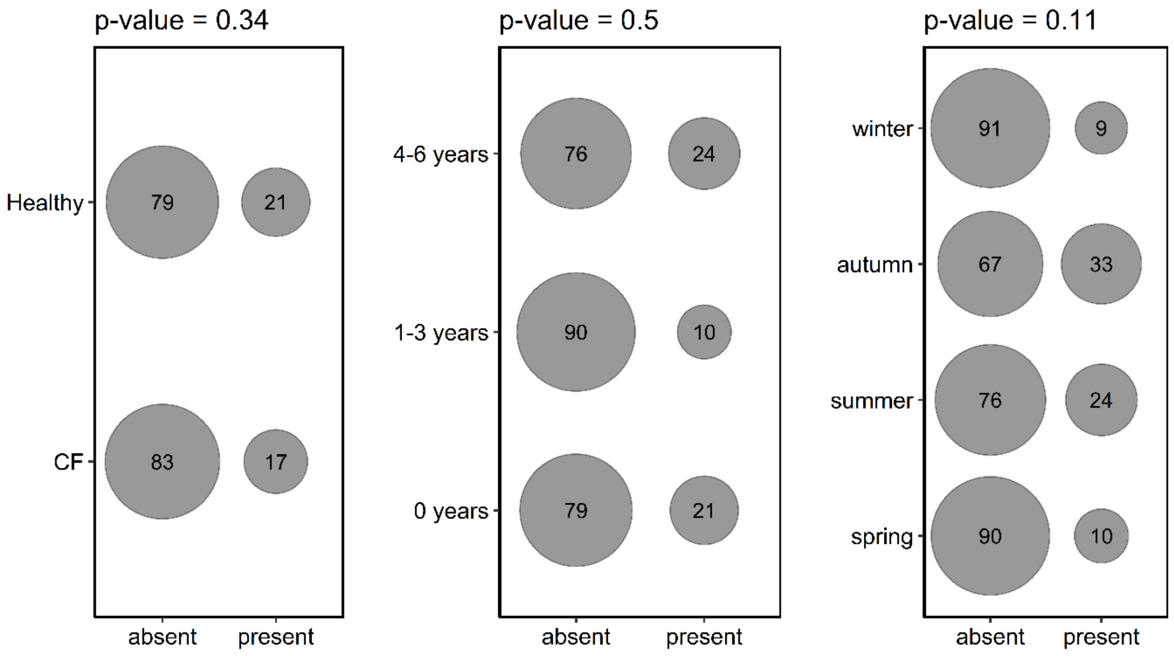

Supplementary Figure 6. Association of *P. aeruginosa*-DNA detection with further clinical and environmental metadata. (**Left**) Proportion of cough swabs (in percentage) from healthy and CF children, which were *P. aeruginosa*-DNA positive (present) or negative (absent). (**Centre**) Number of *P. aeruginosa*-DNA positive (present) and negative (absent) children (in percentage) per age group. (**Right**) Percentage of *P. aeruginosa*-DNA positive (present) and (negative) cough swabs collected at different seasons. The three variables were not associated with significantly increased or decreased *P. aeruginosa*-DNA detection. Fisher's exact test was employed for statistical evaluation of count data with small sample sizes.

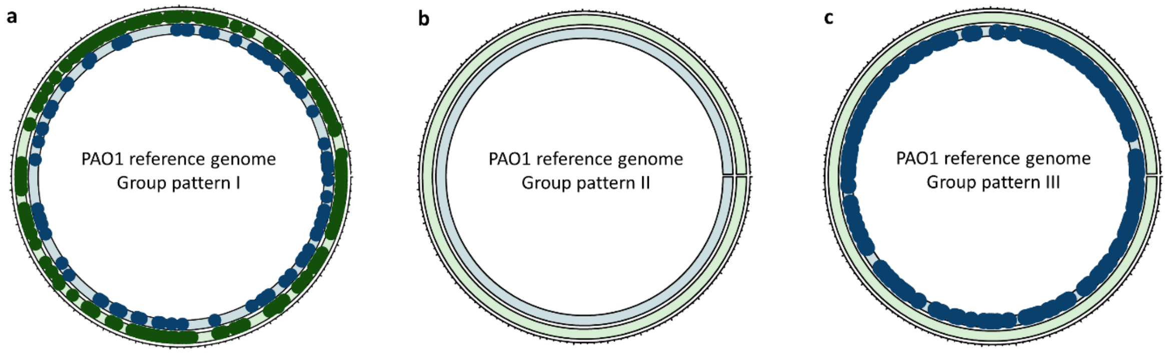

Supplementary Figure 7. Patterns of *P. aeruginosa*-DNA detection in the longitudinal cohort. Three CF children from the longitudinal cohort were picked to represent the three different patterns of *P. aeruginosa*-DNA detection in the group. Each construct was made of two rings, where the outer green ring represents the alignment of *P. aeruginosa* reads (dots) towards the *P. aeruginosa* reference genome (PAO1) in the cough swab obtained at time point one. The inner blue ring shows the alignment of reads towards the reference genome in the sample collected at time point two. (**a**) *P. aeruginosa*-DNA was detected in both consecutive samples of the patient, who remained *P. aeruginosa*-negative in culture diagnostics. (**b**) *P. aeruginosa*-DNA was never detected and the patient remained *P. aeruginosa*-negative in culture. (**c**) *P. aeruginosa*-DNA was absent in the first sample but detected in the second sample. At the second time point, the patient was *P. aeruginosa*-positive in culture for the first time.

Supplementary Tables

Supplementary Table 1. Clinical data of CF patients (cross-sectional CF cohort and longitudinal CF cohort).

Supplementary Table 2. Median relative abundance of core species in healthy and CF children across different age groups. The Mann-Whitney U test was approached for statistical comparison with the corresponding effect size (r) and the confidence intervals of the effect size (CI).

Supplementary Table 3. Variables contributing to the variance observed during the principal component analysis.

Supplementary Table 4. Non-metric multidimensional scaling based on Bray-Curtis dissimilarity matrices of CF patients.

Supplementary Table 5. Programmes for cell lysis (programme 1) and DNA fragmentation (programme 2) using the Covaris S220 Focused-ultrasonicator.

Supplementary Table 1. Clinical data of CF patients (cross-sectional CF cohort and longitudinal CF cohort)

Cross-sectional CF cohort

Patient	Sample	CF diagnosis (months/year)	Diagnosis	Sample taken	Age (in months)	Height (in cm)	Weight (in kg)	BMI (in kg/ m^2)	FVC (Z score)	FEV1 (Z score)	LCI	Sex	Antimicrobial therapy	*CFTR* mutation	Class of mutation **	Pancreatic state
EMCF01	1	04/2013	Gastrointestinal and/or pulmonary symptoms	04.03.2019	75	114	19.9	15.3	0.5	1.2	6.3	m	yes *	p.Phe508del/c.1234delG	II/I	PI
EMCF02	1	08/2015	Meconium ileus	18.02.2019	44	102	17.6	16.9	-0.4	-0.5	9.1	m	none	p.Phe508del/c.1585-1G>A	II/I	PI
EMCF03	1	08/2013	Family history	05.03.2019	72	119	20.9	14.8	0.1	0.3	8.0	f	none	p.Phe508del/p.Phe508del	II/II	PI
EMCF04	1	11/2014	Gastrointestinal and/or pulmonary symptoms	04.02.2019	54	109	16	13.5	-0.1	-0.2	7.3	f	none	p.Phe508del/p.Leu1335Pro	II/II	PI
EMCF05	1	06/2016	Gastrointestinal and/or pulmonary symptoms	08.03.2019	37	90	12.6	15.6	NA	NA	NA	m	none	p.Phe508del/p.Phe508del	II/II	PI
EMCF06	1	11/2018	Gastrointestinal and/or pulmonary symptoms	08.03.2019	38	97	14.7	15.6	0	0	8.8	m	none	No mutations in coding region	V/V	PS
EMCF09	1	09/2015	Family history	15.02.2019	44	99	14.6	15	0	-0.2	9.2	m	none	p.Phe508del/c.3773_3774insT	II/I	PI
EMCF10	1	11/2016	Gastrointestinal and/or pulmonary symptoms	22.01.2019	31	92	15	16.2	NA	NA	NA	f	none	p.Phe508del/p.Phe508del	II/II	PI
EMCF13	1	05/2017	Gastrointestinal and/or pulmonary symptoms	15.04.2019	43	100	16.2	16.2	NA	NA	NA	m	Amoxicillin clavulanic acid	p.Phe508del/c.54-5940_ 273+10250 del21kb	II/I	PI
EMCF14	1	09/2017	Gastrointestinal and/or pulmonary symptoms	28.01.2019	44	89	15	18.9	0	0	8.0	f	none	p.Phe508del/p.Glu92Lys	II/II	PS
EMCF16	1	12/2017	Newborn screening	22.01.2019	14	NA	11.3	NA	NA	NA	NA	m	yes *	p.Phe508del/p.Phe508del	II/II	PI
EMCF17	1	06/2013	Meconium ileus	15.04.2019	70	122	21.6	14.5	-0.5	-0.4	7.4	m	none	p.Phe508del/c.1585-1G>A	II/I	PI
EMCF18	1	03/2019	Gastrointestinal and/or pulmonary symptoms	26.04.2019	32	81	12.4	18.9	0.5	0.3	6.8	f	Cefuroxime	p.Phe508del/p.Phe508del	II/II	PI
EMCF19	1	04/2013	Gastrointestinal and/or pulmonary symptoms	29.04.2019	73	119	20.5	14.5	1	-0.5	7.5	m	none	p.Phe508del/p.Asp1152His	II/IV	PS
EMCF21	1	04/2013	Gastrointestinal and/or pulmonary symptoms	04.02.2019	78	106	16.8	15	-3.5	-2.4	13.6	f	Cefpodoxime, Amoxicillin clavulanic acid	c.579+1G>T/c.54-5940_273+10250del21kb	II/I	PI
EMCF22	1	07/2014	Meconium ileus	03.06.2019	60	110	19.8	16.3	0.4	0.6	5.9	m	none	p.Phe508del/p.Gly542Ter	II/I	PI
EMCF25	1	12/2013	Gastrointestinal and/or pulmonary symptoms	28.06.2019	69	115	20.4	15.5	-0.1	0	7.9	f	Tobramycin	p.Phe508del/no second mutation in coding region	II/V	PI
EMCF26	1	06/2014	Gastrointestinal and/or pulmonary symptoms	29.07.2019	66	108	15.6	13.4	-2.8	-3.7	12.8	f	yes *	p.Leu671Ter/p.Arg1066Cys	II/II	PI
EMCF27	1	11/2015	Family history	18.07.2019	47	101	17	16.7	0	0.1	11.0	m	none	p.Phe508del/p.Phe508del	II/II	PI
EMCF28	1	06/2017	Newborn screening	19.07.2019	25	91	12.5	15.1	NA	NA	NA	m	none	p.Phe508del/p.Phe508del	II/II	PI
EMCF29	1	04/2016	Meconium ileus	19.07.2019	40	97	13.8	14.8	0.1	0.2	8.8	m	none	p.Phe508del/p.Gln1411Ter	II/I	PI
EMCF30	1	02/2015	Gastrointestinal and/or pulmonary symptoms	05.08.2019	79	123	24	15.9	0.6	1.4	9.9	m	Cefuroxime	p.Phe508del/p.Phe508del	II/II	PI
EMCF31	1	06/2017	Newborn screening	12.08.2019	26	92	14.9	17.6	NA	NA	NA	m	none	p.Phe508del/p.Ala1087Pro	II/II	PS
EMCF32	1	06/2014	Gastrointestinal and/or pulmonary symptoms	23.08.2019	78	110	18.6	15.4	-0.1	-0.1	8.3	f	yes *	p.Phe508del/p.Phe508del	II/II	PI

EMCF33	1	10/2015	Gastrointestinal and/or pulmonary symptoms	06.09.2019	52	104	16.8	15.5	NA	NA	4	m	none	p.Phe508del/p.Tyr1092Ter	II/I	PI
EMCF34	1	08/2016	Family history	13.09.2019	53	104	16.1	14.9	0	0.1	6.5	m	none	p.Gly542Ter/p.Asp1152His	I/IV	PS
EMCF35	1	11/2015	Gastrointestinal and/or pulmonary symptoms	13.09.2019	76	113	20.2	15.8	-1.5	-1.5	7.4	m	none	p.Gly542Ter/p.Asp1152His	I/IV	PS
EMCF36	1	02/2016	Gastrointestinal and/or pulmonary symptoms	23.09.2019	82	118	21.3	15	-0.5	-0.8	10.3	f	none	p.Phe508del/p.Phe508del	II/II	PI
EMCF37	1	04/2015	Gastrointestinal and/or pulmonary symptoms	08.11.2019	69	119	23.1	16.3	1.5	0.8	6.8	m	none	p.Phe508del/p.Arg117His	II/IV	PS
MCF13	1	04/2019	Newborn screening	28.10.2019	7	73	9.12	17.1	NA	NA	NA	m	none	p.Phe508del/p.Gly85Glu	II/II	PI
Longitudinal CF cohort																
Patient	Sample	CF diagnosis (months/year)	Diagnosis	Sample taken	Age (in months)	Height (in cm)	Weight (in kg)	BMI (in kg/ m^2)	FVC (Z score)	FEV1 (Z score)	LCI	Sex	Antimicrobial therapy	*CFTR* mutation	Class of mutation **	Pancreatic state
MCF01	1	10/2018	Newborn screening	06.11.2018	1	52	3.9	14.4	NA	NA	NA	f	none	p.Phe508del/p.Phe508del	II/II	PI
MCF01	2	10/2018	Newborn screening	04.12.2018	2	56	5	15.9	NA	NA	NA	f	none	p.Phe508del/p.Phe508del	II/II	PI
MCF01	3	10/2018	Newborn screening	11.01.2019	3	61	5.93	15.9	NA	NA	NA	f	none	p.Phe508del/p.Phe508del	II/II	PI
MCF01	4	10/2018	Newborn screening	22.03.2019	5	66	7.8	17.9	NA	NA	NA	f	none	p.Phe508del/p.Phe508del	II/II	PI
MCF01	5	10/2018	Newborn screening	24.05.2019	7	71	9	17.9	NA	NA	NA	f	none	p.Phe508del/p.Phe508del	II/II	PI
MCF01	6	10/2018	Newborn screening	23.07.2019	9	72	10.1	19.5	NA	NA	NA	f	none	p.Phe508del/p.Phe508del	II/II	PI
MCF01	7	10/2018	Newborn screening	08.11.2019	13	78	10.7	17.6	NA	NA	NA	f	Cefuroxime	p.Phe508del/p.Phe508del	II/II	PI
MCF02	1	11/2018	Newborn screening	13.11.2018	0	NA	4.13	NA	NA	NA	NA	f	none	p.Phe508del/c.54-5940_ 273+10250 del21kb	II/I	PI
MCF02	2	11/2018	Newborn screening	21.01.2019	3	68	7.35	15.9	NA	NA	NA	f	none	p.Phe508del/c.54-5940_ 273+10250 del21kb	II/I	PI
MCF02	3	11/2018	Newborn screening	27.05.2019	7	73	9.28	17.4	NA	NA	NA	f	none	p.Phe508del/c.54-5940_ 273+10250 del21kb	II/I	PI
MCF02	4	11/2018	Newborn screening	26.08.2019	10	78	11.58	19	NA	NA	NA	f	none	p.Phe508del/c.54-5940_ 273+10250 del21kb	II/I	PI
MCF04	1	11/2016	Newborn screening	07.12.2018	26	87	12.4	16.4	NA	NA	NA	m	none	c.2657+5G>A/c.1545_1546delTA	V/I	PS
MCF04	2	11/2016	Newborn screening	08.03.2019	29	NA	13.1	NA	NA	NA	NA	m	none	c.2657+5G>A/c.1545_1546delTA	V/I	PS
MCF05	1	11/2016	Newborn screening	15.01.2019	26	89	12.2	15.4	NA	NA	NA	m	Ciprofloxacin, Colistin	p.Phe508del/p.Phe508del	II/II	PI
MCF05	2	11/2016	Newborn screening	09.04.2019	29	90	13.3	16.4	NA	NA	NA	m	Colistin	p.Phe508del/p.Phe508del	II/II	PI
MCF06	1	12/2016	Newborn screening	11.01.2019	26	84	11.8	16.7	NA	NA	NA	f	Cefadroxil	c.2052_2053insA/c.2989-2A>G	I/I	PI
MCF06	2	12/2016	Newborn screening	26.04.2019	29	86	12.3	16.6	NA	NA	NA	f	Cefadroxil	c.2052_2053insA/c.2989-2A>G	I/I	PI
MCF06	3	12/2016	Newborn screening	16.08.2019	33	90	12.8	15.8	NA	NA	NA	f	none	c.2052_2053insA/c.2989-2A>G	I/I	PI

MCF06	4	12/2016	Newborn screening	15.11.2019	36	91	12.9	15.6	NA	NA	NA	f	none	c.2052_2053insA/c.2989-2A>G	I/I	PI
MCF07	1	03/2018	Newborn screening	12.02.2019	11	74	9	16.4	NA	NA	NA	m	yes *	p.Phe508del/p.Tyr1092Ter	II/I	PI
MCF07	2	03/2018	Newborn screening	21.05.2019	14	79	9.8	15.7	NA	NA	NA	m	none	p.Phe508del/p.Tyr1092Ter	II/I	PI
MCF07	3	03/2018	Newborn screening	20.08.2019	17	80	10.5	16.4	NA	NA	NA	m	none	p.Phe508del/p.Tyr1092Ter	II/I	PI
MCF08	1	03/2019	Newborn screening	12.03.2019	1	58	4.9	14.8	NA	NA	NA	f	none	p.Ser466Ter(TAG)/p.Gly85Glu	II/I	PI
MCF08	2	03/2019	Newborn screening	29.05.2019	3	NA	5.9	NA	NA	NA	NA	f	none	p.Ser466Ter(TAG)/p.Gly85Glu	II/I	PI
MCF08	3	03/2019	Newborn screening	20.08.2019	6	70	6.7	13.7	NA	NA	NA	f	Cefuroxime, Amoxicillin clavulanic acid	p.Ser466Ter(TAG)/p.Gly85Glu	II/I	PI
MCF09	1	02/2018	Newborn screening	18.03.2019	20	82	10.2	15.2	NA	NA	NA	m	none	p.Glu92Lys/p.Glu92Lys	II/II	PS
MCF09	2	02/2018	Newborn screening	22.07.2019	24	88	10.9	14.1	NA	NA	NA	m	none	p.Glu92Lys/p.Glu92Lys	II/II	PS
MCF09	3	02/2018	Newborn screening	30.09.2019	26	89	11.2	14.1	NA	NA	NA	m	none	p.Glu92Lys/p.Glu92Lys	II/II	PS
MCF10	1	12/2017	Newborn screening	29.03.2019	16	83	10.8	15.7	NA	NA	NA	m	Cefuroxime	p.Phe508del/c.42delA	II/I	PI
MCF10	2	12/2017	Newborn screening	29.10.2019	23	110	17.1	14.2	0.7	-0.8	9.9	m	Penicillin G	p.Phe508del/c.42delA	II/I	PI
MCF11	1	12/2017	Newborn screening	15.04.2019	17	86	12.4	16.8	NA	NA	NA	m	yes *	p.Phe508del/p.Phe508del	II/II	PI
MCF11	2	12/2017	Newborn screening	22.07.2019	20	90	13	16	NA	NA	NA	m	none	p.Phe508del/p.Phe508del	II/II	PI
MCF11	3	12/2017	Newborn screening	04.11.2019	24	92	14	16.5	NA	NA	NA	m	Tobramycin	p.Phe508del/p.Phe508del	II/II	PI
MCF12	1	09/2018	Newborn screening	26.04.2019	12	81	10.5	16	NA	NA	NA	m	none	p.Phe508del/p.Phe508del	II/II	PI
MCF12	2	09/2018	Newborn screening	23.08.2019	16	83	11	16	NA	NA	NA	m	none	p.Phe508del/p.Phe508del	II/II	PI
MCF12	3	09/2018	Newborn screening	15.11.2019	18	NA	12.1	NA	NA	NA	NA	m	none	p.Phe508del/p.Phe508del	II/II	PI

*Pharmaceutical active ingredient was unknown

Zielenski, J. & Tsui, L. C. Cystic fibrosis: genotypic and phenotypic variations. *Annu. Rev. Genet.* **29, 777-807 (1995).

Supplementary Table 2. Median relative abundance of core species in healthy and CF children across different age groups. The Mann-Whitney U test was approached for statistical comparison with the corresponding effect size (r) and the confidence intervals of the effect size (CI).

Age group	0 years	0 years	0 years	0 years	0 years	1-3 years	1-3 years	1-3 years	1-3 years	1-3 years	4-6 years	4-6 years	4-6 years	4-6 years	4-6 years
State	Healthy	CF	Mann-Whitney U test			Healthy	CF	Mann-Whitney U test			Healthy	CF	Mann-Whitney U test		
Statistics	median	median	p-value	effect size r	CI (r)	median	median	p-value	effect size r	CI (r)	median	median	p-value	effect size r	CI (r)
Sample size	n = 28	n = 6				n = 9	n=20				n = 15	n = 16			
Genera and core species thereof															
Actinomyces meyeri	0.24	0	0.07	0.32	-0.18 - 0.64	1.44	0.34	0.03	0.41	0.07 - 0.68	**1.71**	**0.17**	**0.0004**	**0.64**	**0.35 - 0.83**
Atopobium parvulum	0.13	0.02	0.09	0.3	-0.01 - 0.56	0.12	0.05	0.12	0.3	-0.05 - 0.63	**0.54**	**0.27**	**0.007**	**0.49**	**0.16 - 0.73**
Campylobacter concisus	0.2	0	0.02	0.43	0.21 - 0.61	1.21	0.83	0.49	0.13	-0.22 - 0.47	**7.47**	**3.45**	**0.004**	**0.5**	**0.18 - 0.75**
Capnocytophaga	0.02	0				0.31	0.79				1.98	0.89			
Capnocytophaga gingivalis	0	0	0.24	0.21	-0.05 - 0.42	0.13	0.33	0.04	0.4	-0.66 - 0.06	1.21	0.54	0.10	0.3	-0.04 - 0.61
Capnocytophaga leadbetteri	0.02	0	0.19	0.24	-0.16 - 0.5	0.18	0.46	0.41	0.16	-0.53 - 0.24	0.77	0.35	0.03	0.38	0.04 - 0.65
Corynebacterium argentoratense	0	0	1	0	-0.39 - 0.28	**0.02**	**0**	**0.0006**	**0.64**	**0.31 - 0.89**	0.01	0	0.14	0.27	-0.05 - 0.59
Eubacterium sulci	0.02	0	0.04	0.37	0.13 - 0.58	0.13	0.23	0.34	0.18	-0.21 - 0.54	**2.84**	**0.43**	**0.001**	**0.6**	**0.32 - 0.8**
Fusobacterium periodonticum	0.02	0	0.16	0.25	-0.04 - 0.5	0.48	1.83	0.65	0.09	-0.27 - 0.46	**14.22**	**2.45**	**0.002**	**0.56**	**0.28 - 0.76**
Haemophilus	1.26	0.02				4.63	16.08				7.03	13.15			
Haemophilus influenzae	0.72	0.02	0.82	0.04	-0.4 - 0.43	2.08	6.62	0.98	0.01	-0.43 - 0.38	3.07	3.21	0.86	0.04	-0.33 - 0.4
Haemophilus parainfluenzae	0.54	0	0.04	0.36	-0.02 - 0.63	2.55	9.46	0.69	0.08	-0.49 - 0.34	3.96	9.94	0.80	0.05	-0.42 - 0.32
Neisseria	0.3	0				4.4	3.58				1.55	3.54			
Neisseria gonorrhoeae	0.04	0	0.15	0.25	-0.17 - 0.53	0.68	0.45	0.91	0.03	-0.32 - 0.37	0.15	0.45	0.92	0.02	-0.34 - 0.37
Neisseria lactamica	0.13	0	0.17	0.24	-0.24 - 0.55	0.86	0.79	0.76	0.06	-0.29 - 0.4	0.3	0.33	0.31	0.18	-0.16 - 0.52
Neisseria meningitidis	0.08	0	0.22	0.22	-0.19 - 0.51	1.28	0.64	0.62	0.1	-0.27 - 0.42	0.25	0.44	0.81	0.05	-0.35 - 0.4
Neisseria mucosa	0.05	0	0.08	0.31	-0.03 - 0.59	1.58	1.7	0.45	0.14	-0.48 - 0.16	0.85	2.32	0.32	0.18	-0.53 - 0.18
Prevotella	1.32	0				8.63	4.64				14.87	18.01			
Prevotella jejuni	0.16	0	0.04	0.37	0.12 - 0.56	0.71	0.5	0.17	0.26	-0.09 - 0.57	3.89	1.87	0.16	0.26	-0.08 - 0.57
Prevotella melaninogenica	1.16	0	0.02	0.43	0.18 - 0.63	7.92	4.14	0.94	0.02	-0.32 - 0.33	10.98	16.14	0.86	0.04	-0.32 - 0.38
Rothia	15.98	9.58				35.3	40.7				15.53	18.48			
Rothia aeria	**0.07**	**0.02**	**0.004**	**0.51**	**0.28 - 0.69**	0.28	0.48	0.89	0.03	-0.38 - 0.32	0.31	0.27	0.59	0.1	-0.45 - 0.27

Rothia mucilaginosa	15.91	9.56	0.14	0.26	-0.16 - 0.56	35.06	40.18	0.80	0.05	-0.4 - 0.31	15.22	18.21	0.52	0.12	-0.44 - 0.28
Streptococcus	76.61	88.64				39.41	28.12				18.96	28.12			
Streptococcus equinus	2.39	6.86	0.21	0.23	-0.56 - 0.25	6.43	2.5	0.22	0.24	-0.13 - 0.55	2.01	1.16	0.61	0.1	-0.46 - 0.29
Streptococcus mitis	41.97	36.18	0.94	0.02	-0.32 - 0.29	4.96	8.55	0.41	0.16	-0.21 - 0.5	3.35	2.93	0.37	0.16	-0.18 - 0.5
Streptococcus oralis	3.12	3.11	0.71	0.07	-0.3 - 0.49	2.05	1.91	0.94	0.02	-0.33 - 0.37	1.29	1.13	0.98	0.01	-0.36 - 0.36
Streptococcus parasanguinis	7.09	13.25	0.19	0.23	-0.11 - 0.49	14.67	3.27	0.05	0.37	0.03 - 0.66	5.97	17.23	0.03	0.38	-0.66 - 0.05
Streptococcus pneumoniae	7.75	4.31	0.74	0.06	-0.34 - 0.44	2.83	3.36	0.25	0.22	-0.16 - 0.54	1.49	1.28	0.53	0.12	-0.26 - 0.46
Streptococcus pseudopneumoniae	12.58	16.76	0.75	0.06	-0.37 - 0.28	5.57	5.92	0.53	0.12	-0.25 - 0.47	2.4	1.41	0.20	0.23	-0.14 - 0.56
Streptococcus salivarius	1.28	7.93	0.63	0.09	-0.56 - 0.41	2.7	2.04	0.20	0.24	-0.1 - 0.54	1.9	2.35	0.89	0.03	-0.33 - 0.36
Streptococcus sanguinis	0.43	0.24	0.86	0.04	-0.37 - 0.46	0.2	0.57	0.25	0.22	-0.56 - 0.14	0.37	0.48	0.37	0.16	-0.49 - 0.2
Streptococcus thermophilus	0.6	0.81	0.80	0.05	-0.55 - 0.44	0.27	0.15	0.21	0.24	-0.12 - 0.54	0.18	0.15	0.98	0.01	-0.38 - 0.34
Veillonella	3.3	0.92				3.59	2.7				13.29	11.05			
Veillonella atypica	**2.6**	**0.8**	**0.009**	**0.44**	**0.14 - 0.66**	1.78	1.31	0.25	0.22	-0.14 - 0.53	11.48	9.23	0.89	0.03	-0.39 - 0.33
Veillonella parvula	0.7	0.12	0.02	0.42	0.09 - 0.67	1.81	1.39	0.35	0.18	-0.17 - 0.49	1.81	1.82	0.25	0.21	-0.54 - 0.16

Significant differences in relative abundance between samples from healthy and CF children are highlighted in bold font. The significance level for statistical testing was 0.01.

Supplementary Table 3. Variables contributing to the variance observed during the principal component analysis

Variable	Dim1	Dim2	Cos2[1]	Contrib[2]
Streptococcus oralis	-0.9237222	0.29624120	0.94	4.8
Rothia mucilaginosa	-0.9235098	0.27982962	0.93	4.7
Veillonella parvula	-0.9574001	0.10577277	0.93	4.7
Streptococcus parasanguinis	-0.9083232	0.29939834	0.91	4.7
Fusobacterium periodonticum	-0.8755037	-0.36904014	0.90	4.6
Actinomyces meyeri	-0.9410270	0.10096403	0.90	4.6
Streptococcus mitis	-0.9153348	0.22133461	0.89	4.5
Streptococcus sanguinis	-0.9256633	0.15124909	0.88	4.5
Neisseria gonorrhoeae	-0.9310432	-0.10168723	0.88	4.5
Neisseria meningitidis	-0.9278126	-0.11361306	0.87	4.4
Neisseria mucosa	-0.8998410	-0.21601130	0.86	4.4
Campylobacter concisus	-0.9044078	-0.17217491	0.85	4.3
Streptococcus salivarius	-0.8486559	0.35656604	0.85	4.3
Streptococcus pneumoniae	-0.8835631	0.20674724	0.82	4.2
Veillonella atypica	-0.8642924	0.24112357	0.81	4.1
Streptococcus pseudopneumoniae	-0.8569169	0.18915066	0.77	3.9
Prevotella melaninogenica	-0.6466619	-0.50520991	0.67	3.4
Haemophilus parainfluenzae	-0.7656215	-0.21318570	0.63	3.2
Eubacterium sulci	-0.3071187	-0.67744340	0.55	2.8
Streptococcus equinus	-0.6998067	0.23183883	0.54	2.8
Neisseria lactamica	-0.7097302	-0.06791028	0.51	2.6
Capnocytophaga leadbetteri	-0.3020869	-0.64568919	0.51	2.6
Prevotella jejuni	-0.4279082	-0.54929682	0.48	2.5
Rothia aeria	-0.6744211	-0.02830531	0.46	2.3
Capnocytophaga gingivalis	-0.3106266	-0.56249328	0.41	2.1

[1] the squared coordinates = (eigenvectors * standard deviations of component)^2

[2] contribution of the variable to the principal components in percentage (cos2 of variable * 100 / cos2 of component)

Supplementary Table 4. Non-metric multidimensional scaling based on Bray-Curtis dissimilarity matrices of CF patients.*		
Variable	Goodness of fit, r2	Goodness of fit, p
Pancreatic status (PI vs PS)	0.009	0.80
Class of mutation (II/I; II/II; IV/other and V/other)	0.11	0.81
Antimicrobial therapy	0.05	0.29
Pseudomonas aeruginosa-DNA	0.06	0.25
Staphylococcus aureus-DNA	0.07	0.25
Age group	0.02	0.53
Body weight	0.04	0.67
Body height	0.06	0.57
BMI	0.35	0.02 *
FVC (Z-score)	0.17	0.17
LCI	0.19	0.13
* A good representation in reduced dimensions was observed (stress = 0.07). The significance of known factors fitted to the ordination was assessed using a permutation test (n = 999, R vegan package, envfit).		

Supplementary Table 5. Programmes for cell lysis (programme 1) and DNA fragmentation (programme 2) using the Covaris S220 Focused-ultrasonicator.
Programme 1<br>Required run time: 3 min, 30 sec<br>Temperature: 5.0 – 8.0 °C<br>Begin repeat (6 x)<br>Treatment (5 sec)<br>Peak power = 200.0, duty factor = 2.0, cycles/burst = 100<br>Treatment (30 sec)<br>Peak power = 275.0, duty factor = 5.0, cycles/burst = 100<br>End repeat
Programme 2<br>Required run time: 6 min<br>Temperature: 5.0 – 8.0 °C<br>Treatment (360 sec)<br>Peak power = 175.0, duty factor = 10.0, cycles/burst = 200
