# Supplementary material for: The human respiratory tract microbial community structures in healthy and cystic fibrosis infants
Source: NPJ Biofilms Microbiomes. 2020 Dec 15;6:61. doi: 10.1038/s41522-020-00171-7 (PMC7738502; doi:10.1038/s41522-020-00171-7)
Supplement: Supplementary file 2 — Reporting Summary [file 41522_2020_171_MOESM2_ESM.pdf]

nature research

Corresponding author(s): Prof. Dr. Dr. Burkhard Tümmler

Last updated by author(s): Nov 8, 2020

Reporting Summary

Nature Research wishes to improve the reproducibility of the work that we publish. This form provides structure for consistency and transparency in reporting. For further information on Nature Research policies, see our Editorial Policies and the Editorial Policy Checklist.

Statistics

For all statistical analyses, confirm that the following items are present in the figure legend, table legend, main text, or Methods section.

n/a	Confirmed	
☐	☒	The exact sample size (*n*) for each experimental group/condition, given as a discrete number and unit of measurement
☐	☒	A statement on whether measurements were taken from distinct samples or whether the same sample was measured repeatedly
☐	☒	The statistical test(s) used AND whether they are one- or two-sided<br>*Only common tests should be described solely by name; describe more complex techniques in the Methods section.*
☐	☒	A description of all covariates tested
☐	☒	A description of any assumptions or corrections, such as tests of normality and adjustment for multiple comparisons
☐	☒	A full description of the statistical parameters including central tendency (e.g. means) or other basic estimates (e.g. regression coefficient) AND variation (e.g. standard deviation) or associated estimates of uncertainty (e.g. confidence intervals)
☐	☒	For null hypothesis testing, the test statistic (e.g. *F*, *t*, *r*) with confidence intervals, effect sizes, degrees of freedom and *P* value noted<br>*Give P values as exact values whenever suitable.*
☒	☐	For Bayesian analysis, information on the choice of priors and Markov chain Monte Carlo settings
☐	☒	For hierarchical and complex designs, identification of the appropriate level for tests and full reporting of outcomes
☐	☒	Estimates of effect sizes (e.g. Cohen's *d*, Pearson's *r*), indicating how they were calculated

Our web collection on statistics for biologists contains articles on many of the points above.

Software and code

Policy information about availability of computer code

Data collection

No software was used for data collection

Data analysis

Metagenome pipelines
Wochenende (version 1.1, https://github.com/MHH-RCUG/Wochenende)
Centrifuge (version 1.0.4)
Metaphlan2 (version 2.96.1)

Reference databases
Wochenende (https://drive.google.com/open?id=1gl6KiY0gOZiz45ulZaWQCLS6A6Su_hrX)
Centrifuge (default)
Metaphlan2 (default)

Data analysis
RStudio (version 3.5.3, platform: x86_64-w64-mingw32/x64 (64-bit))
Packages in R (vegan 2.5-5, rcompanion 2.3.0, ggplot2 3.2.1., corrplot 0.84, conover.test 1.1.5, dplyr 0.8.3, cluster 2.0.7, tidyverse 1.2.1, ggendro 0.1-20)
Gephi (version 0.9.2, https://gephi.org/)

Further software
Raspir (version 1.0), available from https://github.com/mmpust/raspir

For manuscripts utilizing custom algorithms or software that are central to the research but not yet described in published literature, software must be made available to editors and reviewers. We strongly encourage code deposition in a community repository (e.g. GitHub). See the Nature Research guidelines for submitting code & software for further information.

Data

Policy information about availability of data

All manuscripts must include a data availability statement. This statement should provide the following information, where applicable:

- Accession codes, unique identifiers, or web links for publicly available datasets
- A list of figures that have associated raw data
- A description of any restrictions on data availability

The microbial sequencing data are stored in the European Nucleotide Archive (study accession number PRJEB38221).
Absolute abundance estimations of species per sample, metadata and R scripts are available from https://github.com/mmpust/airway-metagenome-infants
The reference database for the read alignment process is publicly available from https://drive.google.com/open?id=1gl6KiY0gOZiz45ulZaWQCLS6A6Su_hrX

Field-specific reporting

Please select the one below that is the best fit for your research. If you are not sure, read the appropriate sections before making your selection.

☒ Life sciences ☐ Behavioural & social sciences ☐ Ecological, evolutionary & environmental sciences

For a reference copy of the document with all sections, see nature.com/documents/nr-reporting-summary-flat.pdf

Life sciences study design

All studies must disclose on these points even when the disclosure is negative.

Sample size

The study includes a cohort of newborns and infants with cystic fibrosis (CF). Sample collection was performed only at one CF center in Germany. The sample size was therefore, naturally limited (n = 41). However, we on purpose decided to not rely on a multi-center study, because two of the most flawed aspects in respiratory tract metagenome studies are the sampling collection procedure and DNA background contamination. From previous experiments it became evident that swabs are useless if performed by unexperienced staff. An obligate cough of the patient has to be induced to investigate the lower airways. Also, the latent period between sample collection and quick freezing should be less than 30 seconds and cold chains have to be maintained at all times, otherwise the microbial community structure is distorted. It was shown that DNA contamination varies depending on the environment of sample collection and laboratories. By setting-up a one-center study, we were able to control contamination, maintain cold chains and freeze samples within 30 seconds. We established a standard cleaning procedure of the laboratory environment which was uniformly conducted before sample processing. Before sample processing, aliquots for use in all biological samples and negative controls were prepared simultaneously and stored as required, so that all samples were treated with the same kits by lot number Additionally, all swab collections were performed by same trained team of pneumologists. If the participants did not cough during sample collection, the procedure was repeated. However, the sample size was low. Therefore, we calculated effect sizes, which are sample size independent and their corresponding confidence intervals. We used different clustering methods and metagenomics pipelines to confirm the real biological patterns.

Data exclusions

One infant in the CF cohort with a positive newborn-screening result was excluded from downstream analysis because the diagnosis was later found to be inconclusive (CF-SPID).

Replication

The sample collection and wet-lab procedures were not repeated due to the limited biologial materials. However, we strongly recommend to repeat the study in geographically-distant CF care settings.

Randomization

The allocation of study participants in our study was not random. There were two groups (CF and healthy).

Blinding

After the sample collection procedure, all samples were anonymised and received a neutral participant ID. During the wet-lab procedure and unsupervised clustering analysis, it was not known if a sample comes from a CF or healthy study participant.

Reporting for specific materials, systems and methods

We require information from authors about some types of materials, experimental systems and methods used in many studies. Here, indicate whether each material, system or method listed is relevant to your study. If you are not sure if a list item applies to your research, read the appropriate section before selecting a response.

Materials & experimental systems

n/a	Involved in the study
☒	☐ Antibodies
☒	☐ Eukaryotic cell lines
☒	☐ Palaeontology and archaeology
☒	☐ Animals and other organisms
☐	☒ Human research participants
☒	☐ Clinical data
☒	☐ Dual use research of concern

Methods

n/a	Involved in the study
☒	☐ ChIP-seq
☒	☐ Flow cytometry
☒	☐ MRI-based neuroimaging

Human research participants

Policy information about studies involving human research participants

Population characteristics

We collected cough swabs from 41 patients with CF and 52 healthy controls between zero to six years of age. At sampling, CF children had a median age of 26 months (0-82, median absolute deviation = 25.2) and healthy children were slightly younger with a median age of 11 months (1-75, median absolute deviation = 11.9). Age (in months) at sampling was hence different between the CF and healthy cohort (Wilcoxon p-value = 0.003, effect size r = 0.31, CI = 0.12 -0.48). However, the gender distribution of healthy and CF cohort was not different (Fisher's exact test for count data, p-value > 0.05). All CF samples were collected at the CF outpatient clinic at MHH. Healthy samples were collected at Kindergartens (33 %), local paediatricians (31 %) and parent-child groups (36 %). Most of the CF children in the study were either diagnosed due to gastrointestinal and/or pulmonary symptoms (43 %) or the CF newborn screening (37 %) and most of the CF children were pancreatic insufficient (80 %).

Recruitment

All CF participants were regularly seen and monitored by CF specialists at the MHH since the age of diagnosis. All parents received an information letter about the background and objective of the shotgun metagenomic study two weeks before the regular examination appointment at MHH. On the day of the examination, the study physician informed parents about the study, the sample collection procedure, data protection rights, data storage procedures and the potential publication of anonymised results. All parents and/or legal guardians gave their written consent.
In the case of healthy participants, local childcare facilities and paediatricians were contacted and their cooperation was requested. Study information was posted publicly on notice boards in the corresponding facilities, group leaders and paediatricians were asked to pre-select interested families. Afterwards, the study physician again informed parents in detail about the procedures, data management and potential publication. All parents and/or legal guardians gave their written consent.

Ethics oversight

The clinical study was approved by the ethics committees of MHH (No. 7674).

Note that full information on the approval of the study protocol must also be provided in the manuscript.
